# Sexual and Gender Diversity in Thailand: Associations with Recalled Childhood Sex-Typed Behavior and Adulthood Occupational Preferences

**DOI:** 10.1007/s10508-025-03121-6

**Published:** 2025-04-24

**Authors:** Francisco R. Gómez Jiménez, Ashley K. Dhillon, Doug P. VanderLaan

**Affiliations:** 1 Centre for Culture and Evolution, Brunel University of London, Uxbridge, UB8 3PH UK; 2 Department of Psychology, University of Toronto Mississauga, Mississauga, ON L5L 1C6 Canada; 3 Child and Youth Psychiatry, Centre for Addiction and Mental Health, Toronto, ON Canada

**Keywords:** Childhood sex-atypical behavior, Occupational preferences, Sexual orientation, *Sao praphet song*, *Toms*, *Dees*

## Abstract

Same-sex attracted individuals report greater levels of sex-atypical childhood behaviors and adulthood occupational preferences when compared with their heterosexual counterparts. While these sexual orientation differences are well established, the extent to which gender-role presentation relates to such differences is unclear. The present study examined recalled childhood sex-(a)typical behaviors (CSAB) and adulthood occupational preferences in a diverse Thai sample (*N* = 1294) of cisgender heterosexual men (*n* = 270) and women (*n* = 280), gay men (*n* = 199), lesbian women (*n* = 56), and unique Thai sexual orientation/gender categories: *sao praphet song* (i.e., feminine-presenting same-sex attracted males; *n* = 166), *toms* (i.e., masculine-presenting same-sex attracted females; *n* = 174), and *dees* (i.e., feminine-presenting females sexually attracted to *toms*; *n* = 149). Gay men and *sao praphet song* reported more CSAB and sex-atypical adulthood occupational preferences than heterosexual men, and *sao praphet song* were more sex-atypical than gay men. *Toms* reported more CSAB and sex-atypical adulthood occupational preferences than heterosexual women, lesbian women, and *dees*, whereas lesbian women were more sex-atypical than heterosexual women and *dees* in childhood but not adulthood. CSAB was associated with sex-atypical adulthood occupational preferences among heterosexual men and all same-sex attracted groups, indicating continuity in gender-role expression development. Overall, this study replicates previous findings indicating greater sex-atypical behaviors and interests during childhood and adulthood among same-sex attracted individuals. It also expands upon prior literature by showing how gender-role presentation relates to these sexual orientation differences among males and females.

**Supplementary Information:**

The online version contains supplementary material available at 10.1007/s10508-025-03121-6.

## Introduction

Same-sex sexual orientation in males and females is often associated with the expression of behaviors and interests that vary from the ones typically exhibited in childhood and adulthood by members of the same sex.^1^ In childhood, sex-typical behaviors can include, for example, a preference for girls as playmates and taking on female personas during imaginary play among females, and preferences for boys as playmates and rough-and-tumble play among males (e.g., Li et al., 2017; Wong & Hines, 2015). During adulthood, occupational preferences can be used as an analogous proxy for sex-typicality. On average, heterosexual men tend to prefer systematizing, thing-oriented occupations (e.g., carpenter, mechanic, engineer), whereas heterosexual women tend to prefer empathizing, people-oriented occupations (e.g., counselor, clinician, elementary schoolteacher; for reviews, see Archer, 2019; Konrad et al., 2000). These adulthood sex differences have been documented in large cross-cultural studies (e.g., Lippa, 2008a, 2010). Similarly, sexual orientation differences in sex-typed behaviors and interests have also been documented across cultures.

Androphilic males (i.e., males who are sexually attracted to other males) report greater female-typical and lower male-typical childhood behaviors when compared with heterosexual men (see Bailey & Zucker, 1995, for a meta-analysis). This pattern has been confirmed with retrospective and prospective studies of cisgender participants in both Euro-American (Cardoso, 2005; Li et al., 2017; Rieger et al., 2008; Singh et al., 2021; Steensma et al., 2013; Wallien & Cohen-Kettenis, 2008; Zucker, 2008) and non-Euro-American contexts (e.g., Besharat et al., 2016; Cardoso, 2009; Gómez Jiménez et al., 2020; Petterson et al., 2017; Sadr-Bazzaz et al., 2024a). Similar studies have examined samples of transgender/gender-diverse (TGD) androphilic males, finding the same pattern of sex-atypical childhood behavior in Euro-American (Singh et al., 2021; Wallien & Cohen-Kettenis, 2008) and non-Euro-American samples (Gómez Jiménez et al., 2020; Roshan et al., 2019; Sadr-Bazzaz et al., 2024a; Semenyna & Vasey, 2016; Whitam, 1983).^2^

These male sexual orientation differences are also reflected in adulthood in the form of elevated preferences for empathizing (i.e., female-typical) occupations and lower levels of preference for systematizing (i.e., male-typical) occupations among male androphiles. Among cisgender androphilic males, these sex-atypical occupational preferences have been documented in both Euro-American (e.g., Ellis et al., 2012; Lippa, 2002, 2005a, 2008a, 2020; VanderLaan et al., 2016) and non-Euro-American cultures (e.g., Gómez Jiménez et al., 2021; Sadr-Bazzaz et al., 2024b; Whitam & Mathy, 1986; Zheng et al., 2011). Among TGD androphilic males, sex-atypical occupational preferences have been mainly documented in non-Euro-American cultures (e.g., Gómez Jiménez et al., 2021; Hart, 1968; Sadr-Bazzaz et al., 2024b; Semenyna & Vasey, 2016; Stief, 2017). Altogether, this research suggests that the expression of sex-atypical childhood behaviors and adulthood occupational preferences are cross-culturally universal correlates of male androphilia regardless of whether male androphilia is expressed in a cisgender or TGD form.

Similar research among gynephilic females (i.e., females who are sexually attracted to other females) is relatively rare and mostly limited to Euro-American cultures, but the available evidence also demonstrates sexual orientation differences in childhood and adulthood sex-atypicality. When compared with heterosexual women, retrospective and prospective research indicates that cisgender (e.g., Bailey & Zucker, 1995; Drummond et al., 2008; Li et al., 2017; Rieger et al., 2008; VanderLaan et al., 2015; Wallien & Cohen-Kettenis, 2008; Whitam, 1987; Zucker, 2008) and TGD (e.g., Drummond et al., 2008; Wallien & Cohen-Kettenis, 2008) gynephilic females exhibit elevated male-typical and lowered female-typical behaviors in childhood. Three studies conducted in non-Euro-American cultures have found similar results among cisgender (Sadr-Bazzaz et al., 2024a; Whitam, 1987) and TGD (Roshan et al., 2019; Sadr-Bazzaz et al., 2024a) gynephilic females.

In adulthood, sex-atypical occupational preferences have been documented among cisgender gynephilic females in Euro-American (e.g., Ellis et al., 2012; Lippa, 2005a, 2008a, 2008b, 2020) and non-Euro-American cultures (e.g., Sadr-Bazzaz et al., 2024b; Whitam, 1987; Zheng et al., 2011). The only study to date to quantitatively explore occupational preferences among transgender gynephilic females was conducted in Iran and found greater sex-atypical preferences among this group relative to cisgender heterosexual women (Sadr-Bazzaz et al., 2024b). Thus, further research is needed from additional cultures to ascertain whether sex-atypical childhood behaviors and adulthood occupational preferences are cross-cultural correlates of female gynephilia.

The consistency of these sexual orientation differences across different cultural contexts suggests that the expression of sex-atypical behaviors among same-sex attracted individuals are, in part, due to biodevelopmental factors that transcend populations. For example, one biological explanation is that exposure and receptivity to sex-atypical levels of sex-steroid hormones during sensitive periods of pre-/peri-natal development “feminize” areas of males’ brains and “masculinize” areas of females’ brains that regulate sexual orientation and its correlated behaviors (e.g., Balthazart, 2016; Bao & Swaab, 2011; Berenbaum & Beltz, 2011, 2016; Breedlove, 2017; Ellis & Ames, 1987; Hines et al., 2015). Consistent with this idea, research on individuals with XY sex chromosomes and complete androgen insensitivity syndrome (CAIS), whose body tissues are not sensitive to the presence of androgens, demonstrates that they experience female-typical development with respect to sexual orientation and childhood behaviors and interests (e.g., Hines et al., 2003; Khorashad et al., 2018). Similarly, research on individuals with XX sex chromosomes and congenital adrenal hyperplasia (CAH), who produce high levels of androgens, has found that such individuals have an elevated likelihood of reporting non-heterosexual orientations (e.g., Berenbaum et al., 2012; Meyer-Bahlburg et al., 2008; Puts et al., 2008) and greater levels of male-typical childhood behaviors and adulthood interests (e.g., Beltz et al., 2011; Berenbaum & Snyder, 1995; Khorashad et al., 2018). Altogether, this research suggests that sexual orientation differences in sex-typed behaviors are partially due to differences in hormone exposure and receptivity during critical periods of brain development.

While male and female sexual orientation differences in sex-atypical behaviors and interests are well established, the differences between cisgender and TGD same-sex attracted individuals have rarely been explored. Across prior studies, cisgender same-sex attracted individuals exhibit a pattern of sex-typed behaviors and interests that tends to be in between heterosexual men and women (e.g., Bailey & Zucker, 1995; Ellis et al., 2012; Lippa, 2005a, 2008a, 2008b, 2020; VanderLaan et al., 2015, 2016; Zheng et al., 2011), whereas TGD same-sex attracted individuals exhibit one that either mirrors or exaggerates the pattern displayed by the other sex (Roshan et al., 2019; Semenyna & Vasey, 2016; VanderLaan et al., 2017). Only a few studies have directly compared the sex-typed behaviors of cisgender and TGD same-sex attracted individuals. With respect to male androphilia, these studies demonstrated that TGD androphilic males exhibit more female-typical childhood sex-typed behaviors (e.g., Gómez Jiménez et al., 2020; Sadr-Bazzaz et al., 2024a; Singh et al., 2021; Wallien & Cohen-Kettenis, 2008) and adulthood occupational preferences (Gómez Jiménez et al., 2021; Sadr-Bazzaz et al., 2024b) than cisgender androphilic males. With respect to female gynephilia, two studies conducted in Iran have similarly found that transgender gynephilic females exhibit greater male-typical childhood sex-typed behaviors (Sadr-Bazzaz et al., 2024a) and adulthood occupational preferences (Sadr-Bazzaz et al., 2024b) than cisgender gynephilic females.

Expanding on the neuroendocrinological explanation of same-sex sexual attraction, researchers have speculated that variability in sex-typed behaviors and interests found among cisgender and TGD same-sex attracted individuals could reflect a “dosage” effect of sex-atypical hormone exposure during development (e.g., VanderLaan et al., 2023). That is, some exposure to sex-atypical levels of hormones during critical periods of brain development would lead to same-sex attraction and some degree of sex-atypicality whereas higher levels would lead to both same-sex sexual attraction and greater sex-atypicality. This could potentially explain why transgender same-sex attracted individuals tend to display greater sex-atypical behaviors and interests than cisgender same-sex attracted individuals among the few studies that have conducted such comparisons (Gómez Jiménez et al., 2020, 2021; Sadr-Bazzaz et al., 2024a, 2024b; Singh et al., 2021; Wallien & Cohen-Kettenis, 2008). Nevertheless, further research is required to determine whether the differences between cisgender and transgender same-sex attracted individuals are consistent across cultures, and whether similar patterns are found among both males and females.

To address this gap in the literature, the present study assessed sex, gender, and sexual orientation differences in childhood and adulthood sex-typed behaviors and interests in Thailand, where sexual and gender diversity is culturally widespread and reputedly accepted (Coome et al., 2020; Sinnott, 2004; Totman, 2003; Winter, 2006, 2013; but also see Gooren et al., 2015; Jackson, 1999; Miedema et al., 2022; Srikummoon et al., 2022, 2023; Winter, 2011). In this culture, sexually and gender-diverse individuals often identify and are identified with unique and widely recognized non-binary genders locally known as *sao praphet song*, *toms*, and *dees*. *Sao praphet song*, which translates to “a second kind of woman,” are transfeminine individuals assigned male at birth and who are most often sexually attracted to cisgender men (Coome et al., 2020; Totman, 2003; Winter, 2006, 2013). *Toms*, which derives from the English word “tomboy,” are transmasculine individuals assigned female at birth and who are most often sexually attracted to either cisgender women or *dees* (Coome et al., 2020; Sinnott, 2004). *Dees*, which derives from the last syllable of the English word “lady,” are birth-assigned females who are feminine with respect to their gender-role presentation—and thus, the term cisgender may apply—and are sexually and romantically involved with *toms* (Coome et al., 2020; Sinnott, 2004). In addition to being described in the literature as sexually attracted toward *toms* (Miedema et al., 2022; Sinnott, 1999), *dees* may also find that *toms* are attentive partners who tend to perform more care-giving than heterosexual men, which can be an appealing aspect of *tom-dee* relationships (Sinnott, 2004).

In addition to these unique non-binary genders, same-sex attracted individuals who display sex-typical gender-role presentation in Thailand may identify as gay men and lesbian women similar to cisgender same-sex attracted individuals in Euro-American cultures (e.g., Coome et al., 2020; Sinnott, 2004; Totman, 2003). Nevertheless, given that gender and sexuality are often conflated in everyday Thai language, both cisgender and TGD same-sex attracted individuals in Thailand are often considered to be *pheet thii saam*, which translates to “third sex/gender” (Miedema et al., 2022; Sinnott, 2004). This rich and widespread diversity of gender and sexual expression in Thailand makes it an ideal culture to explore the ways in which sex, gender-role presentation, and sexual orientation relate to the expression of sex-typed behaviors and interests in childhood and adulthood. While one study found that androphilic men in Thailand recalled more female-typical childhood behaviors than gynephilic men (Cardoso, 2009), the sex-typed behaviors and interests of other *pheet thii saam* groups have not been explored.

Based on the literature reviewed above, we made several predictions. First, we expected to find sex differences between heterosexual men and women in childhood sex-typed behaviors and adulthood occupational preferences. Second, we predicted sexual orientation differences with same-sex attracted individuals displaying greater sex-atypical childhood and adulthood sex-typed behaviors and interests compared with their heterosexual counterparts. Finally, we predicted gender-role presentation differences. Cisgender same-sex attracted individuals (i.e., gay men, lesbian women, and *dees*) were expected to exhibit a pattern of childhood and adulthood sex-typed behaviors and interests that was intermediate between heterosexual men and women. In contrast, we expected that TGD same-sex attracted individuals (i.e., *sao praphet song* and *toms*) would exhibit a pattern of sex-typed behaviors and interests that either mirrored or exaggerated the pattern seen among heterosexual individuals of the other sex.

In addition to examining the influences of sex, gender-role presentation, and sexual orientation on childhood sex-typed behavior and adulthood occupational preferences, the present study also explored whether there is a correlation between these two variables. Prior research has found that recalling elevated levels of sex-atypical behavior in childhood is associated with the expression of sex-atypical occupational preferences in adulthood among heterosexuals and cisgender and TGD same-sex attracted individuals, suggesting that there might be a developmental continuity in the expression of sex-atypicality (Gómez Jiménez et al., 2021; Lippa, 2008b; Sadr-Bazzaz et al., 2024b; Semenyna & Vasey, 2016; VanderLaan et al., 2016). However, most of these studies have focused on androphilic males and only one of these included a sample of transgender gynephilic females (Sadr-Bazzaz et al., 2024b). Thus, the present study expanded upon this literature by examining the association between childhood sex-typed behavior and adulthood occupational preferences among a diverse set of Thai cisgender and TGD same-sex attracted males and females. Given the consistency of the results from previous studies, we expected to find that sex-atypicality in childhood would predict sex-atypicality in adulthood independent of gender identity and sexual orientation.

## Method

### Participants

All participants were recruited between May–July 2017 in Chiang Mai, Thailand, and the surrounding area via a network sampling procedure. Chiang Mai is the largest urban area in the Northern region of Thailand. The researchers approached people in public spaces (e.g., parks, shopping centers, village markets, along the street) to share information about the study and to invite them to participate. Those interested in participating made an appointment to do so and completed study measures in-person. Following participation, they were asked to share information about the study with others, particularly to those who are sexually or gender diverse, who might be interested to participate. This process continued throughout the period of participant recruitment. Participants provided informed written consent prior to taking part in the study. The rate of participation was > 90% among those invited by the study team. All participants received 300 Thai Baht as an honorarium for completing a battery of measures.

A total of 1423 participants were interviewed for the present study. Given that the present study focused on assessing differences in behavior and interests between gynephilic and androphilic males and females, individuals who identified as bisexual (*n* = 65) or reported their birth-assigned sex as ambiguous (*n* = 9) were excluded from the final sample size. Seven individuals who identified as transgender men were excluded given the small group size, and an additional 48 individuals (16 heterosexual men, 5 gay men, 9 *sao praphet song*, 6 *toms*, 3 lesbian women, 4 *dees*, and 5 heterosexual woman) with data outliers (see Statistical Analyses subsection) were excluded. Thus, the final sample size consisted of 270 heterosexual men, 199 gay men, 166 *sao prophet song*, 174 *toms*, 56 lesbian women, 149 *dees*, and 280 heterosexual women.

### Procedure and Measures

Participants were interviewed by a Thai research assistant under the last author’s supervision using standardized questionnaires, which were available in Thai after being translated and back-translated by two fluent Thai-English speakers. A Thai-speaking research assistant was available to answer participants’ questions. Participants were asked to report information regarding their age (in years), average monthly income, and level of education. Participants’ monthly income was coded as either “9999 Thai baht or less” or “10,000 Thai baht or more” and participants’ education was coded as either “Post-secondary not complete” or “Post-secondary complete.”

Participants reported their sex at birth, their current gender identity (specifically, “sex you feel like you are/gender/sexual identity”), and sexual attraction during the previous year.^3^ For sex at birth, participants were asked to select between one of the following: “male/man,” “female/woman,” and “ambiguous/other.” For current sex/gender/sexual identity, participants were asked to select between one of the following: “male/man,” “female/woman,” “gay,” “lesbian,” “bi-woman,” “bi-man,” “*sao praphet song*,” “*tom*,” and “*dee*,” or they could specify another identity. We divided the participants into seven groups based on their current sex/gender/sexual identity and sex assigned at birth: heterosexual men, gay men, *sao prophet song*, *toms*, lesbian women, *dees*, and heterosexual women.

Sexual orientation was assessed by asking participants about their sexual attraction during the last 12 months toward men (including heterosexual, bisexual, and/or gay men), women (including heterosexual, bisexual, and/or lesbian women, as well as *dees*), *sao praphet song*, and *toms*. Participants responded using a seven-point scale ranging from 0 (*none of my sexual attractions*) to 6 (*all of my sexual attractions*). Responses to the four sexual attraction targets needed to sum to 6 to represent all sexual attractions experienced over the past year. As shown in Table 1, participants’ sexual attraction ratings were in alignment with their gender/sexual orientation identities.^4^

**Table 1:** Descriptive statistics for sexual attractions during the previous year by group

Group	*n*	Sexual Attraction Target *M* (*SD*)			
		Men	Women	*Toms*	*Sao Praphet Song*
Heterosexual men	270	0.07 (0.54)	5.72 (0.76)	0.09 (0.34)	0.12 (0.38)
Gay men	198	5.49 (1.13)	0.27 (0.70)	0.03 (0.20)	0.21 (0.82)
*Sao Praphet Song*	166	5.84 (0.69)	0.04 (0.34)	0.09 (0.48)	0.02 (0.19)
*Toms*	174	0.07 (0.32)	5.74 (0.78)	0.09 (0.51)	0.11 (0.39)
Lesbian women	56	0.38 (0.62)	4.54 (1.63)	0.95 (1.49)	0.14 (0.44)
*Dees*	149	0.41 (0.80)	0.44 (0.97)	5.13 (1.31)	0.02 (0.14)
Heterosexual women	277	5.58 (0.90)	0.24 (0.62)	0.16 (0.57)	0.03 (0.17)

For all sexual attraction questions, the possible range for responses was from 0, representing “none of my sexual attractions” to 6, representing “all of my sexual attractions.” Each participant’s responses to the sexual attraction questions had to sum to 6. The target “men” included heterosexual, bisexual, and/or gay men. The target “women” included heterosexual, bisexual, and/or lesbian women, as well as *dees*

Childhood sex-typed behavior was assessed using the Childhood Gender Identity Scale (Bartlett & Vasey, 2006). This scale is derived from the Gender Identity Questionnaire for Children (Johnson et al., 2004)—a parent-report questionnaire used to assess children’s gender expression. The Childhood Gender Identity Scale consists of a childhood female-typical behavior (CFTB) subscale containing six items and a childhood male-typical behavior (CMTB) subscale containing five items. Participants were asked to recall how often they exhibited female-typical (e.g., “put on girls’ makeup or clothes or accessories”) and male-typical behaviors (e.g., “rough and tumble play”) before the age of 12 years using a 5-point scale (1 = “Never” to 5 = “Always/All the time”). Mean scores were calculated from each of the two subscales to obtain recalled CFTB and CMTB subscale scores. This scale and/or approach has been used to assess sex, sexual orientation, and gender-role differences in childhood sex-typed behavior across cultures (e.g., Bartlett & Vasey, 2006; Gómez Jiménez et al., 2020, 2021; Petterson et al., 2017; Sadr-Bazzaz et al., 2024a, 2024b; Semenyna & Vasey, 2016; VanderLaan et al., 2011, 2015, 2016, 2017) and, thus, allows us to provide results that are readily comparable across studies. Cronbach’s alpha coefficients for CMTB (α = 0.89) and CFTB (α = 0.93) were acceptable in the present sample. These alphas were similar to those found in previous studies looking at sexual orientation differences in recalled childhood sex-typed behaviors (e.g., Bartlett & Vasey, 2006; Gómez Jiménez et al., 2020, 2021; Semenyna & Vasey, 2016; VanderLaan et al., 2015, 2016, 2017). A childhood sex-atypical behavior (CSAB) composite score was created for all males (i.e., heterosexual men, gay men, and *sao prophet song*) by subtracting CMTB from CFTB, and for females (i.e., heterosexual women, lesbian women, *dees*, and *toms*) by subtracting CFTB from CMTB. A constant of 4 was added to all scores so the values for CSAB ranged from 0 to 8. Thus, for both male and female participants, a score of 0 indicates no sex-atypical behavior, whereas higher values indicate greater sex-atypical behavior recalled during childhood.

Occupational preferences were evaluated using Lippa’s (2008a, 2008b) measure in which participants rated their interest in the following occupations: car mechanic, clothes designer, constructor, social worker, inventor, dance teacher, carpenter, teacher, electronic engineer, and flower seller/florist. Participants rated the 10 occupations using a seven-point scale (1 = “strongly dislike” to 7 = “strongly like”). Previous research indicated that the odd-numbered items are typically preferred by heterosexual men, whereas the even-numbered items are typically preferred by heterosexual women (Lippa, 2008a, 2010). Thus, responses to the even-numbered items were averaged to create a male-typical occupational preference (MTOP) score and responses to the odd-numbered items were averaged to create a female-typical occupational preference (FTOP) score. This approach has been used to assess sex, sexual orientation, and gender-role differences in occupational preferences across cultures (e.g., Gómez Jiménez et al., 2021; Lippa, 2008a, 2010, 2020; Sadr-Bazzaz et al., 2024b; Semenyna & Vasey, 2016; Zheng et al., 2011) and, thus, allows us to provide results that are readily comparable across studies. Cronbach’s alpha coefficients were acceptable in this sample for FTOP (α = 0.68) and MTOP (α = 0.83). These alphas were similar to those found in previous studies looking at sexual orientation differences in occupational preferences (e.g., Lippa, 2010, 2020; Semenyna & Vasey, 2016; Zheng et al., 2011). Consistent with prior research (Lippa, 1991, 2005b), a male- versus female-typical occupational preference (MF-Occ) score was calculated by subtracting the MTOP scores from FTOP. Thus, positive scores indicate greater female-typical occupational preferences, whereas negative scores indicate greater male-typical occupational preferences.

### Statistical Analyses

Statistical analyses were conducted using R version 4.3.2 (R Core Team, 2022). Data outliers (i.e., *z*-scores ≤ -3.29 and ≥ 3.29) within group were removed (Field, 2024). A reciprocal transformation was used on age due to extreme skewness. To assess the associations between the descriptive variables and the childhood and adulthood sex-typed variables, we conducted Pearson’s *r* correlations for age and point biserial correlations for level of education and weekly income due to the dichotomous nature of these latter two variables. Due to a significant Levene’s test for equality of variance (*p* < .001), group differences in age were assessed using a Welch one-way analysis of variance (ANOVA) and post hoc pairwise comparisons were conducted using the Games-Howell procedure. Group differences in weekly income and level of education were assessed using Chi-square analyses because of the categorical nature of these variables. To determine which groups differed, post hoc pairwise comparisons were conducted following significant Chi-square analyses by using *z*-tests comparing the proportion of weekly income and level of education between groups. The critical alpha for the *z*-tests was adjusted to 0.0024 using the Bonferroni correction given the high number of pairwise comparisons (21 in total).

Group differences in the MTOP and FTOP scores, the average MF-Occ score, the average recalled CFTB and CMTB subscale scores, and the CSAB composite score between Thai heterosexual men, gay men, *sao prophet song*, heterosexual women, lesbian women, *dees*, and *toms* were assessed using one-way ANOVAs. To determine the size and direction of significant omnibus tests, post hoc pairwise comparisons were conducted using the Games-Howell procedure for all variables because of differences in sample sizes and variance between groups (see Results). Unequal-variance Cohen’s *d* statistics were calculated for all pairwise comparisons as $\frac{{\text{M}}_{1} - {\text{M}}_{1}}{\sqrt{\frac{{\text{SD}}_{1}^{2} \, + \text{ } {\text{SD}}_{2}^{2}}{2}}}$ and presented with their bias-corrected and accelerated 95% confidence intervals (Cohen, 1988; Navarro, 2015).

Similar to Gómez Jiménez et al. (2021), multiple linear regression analyses were conducted to assess whether the relationship between childhood sex-atypical behavior and adulthood occupational preferences was independent of sexual orientation and gender identity among males and females separately. To do so, dummy coded variables were created for the male and female gender groups. For males, two dummy coded variables labeled Heterosexual Men vs. Gay Men and Heterosexual Men vs. *Sao Praphet Song* were created. Thus, heterosexual men were the reference group. For females, three dummy coded variables labeled Heterosexual Women vs. Lesbian Women, Heterosexual Women vs. *Dees*, and Heterosexual Women vs. *Toms* were created. Thus, heterosexual women were the reference group. For all analyses of gender expression, critical alpha was set at 0.05—as in prior similar studies (e.g., Gómez Jiménez et al., 2020, 2021; Lippa, 2010, 2020; Semenyna & Vasey, 2016; VanderLaan et al., 2011, 2015, 2016, 2017; Zheng et al., 2011), thus facilitating comparability of findings across cultures.

## Results

Descriptive statistics for the demographic information are presented in Table 2. Significant differences were found for age, *F*(6, 419.18) = 9.42, *p* < .001, weekly income, *χ*^2^(6) = 21.97, *p* = .001, and level of education, χ^2^(6) = 35.04, *p* < .001. With respect to age, heterosexual women were significantly older than *dees* (*p* = .040), lesbian women (*p* = .018), *sao prophet song* (*p* = .004), and gay men (*p* < .001); *toms* were significantly older than *sao prophet song* (*p* = .016), and gay men (*p* < .001); and heterosexual men were significantly older than gay men (*p* < .001). With respect to weekly income, a significantly lower proportion of gay men reported earning 10,000 Baht or more compared with *dees* (*p* < .001) and *toms* (*p* < .001), and a significantly lower proportion of *sao prophet song* reported earning 10,000 Baht or more compared with *dees* (*p* = .002). With respect to level of education, a significantly higher proportion of *toms* reported receiving post-secondary education than heterosexual men (*p* < .001), gay men (*p* < .001), and *sao prophet song* (*p* < .001). Nonetheless, the descriptive variables were not used as covariates in further analyses given that they were only weakly (*r* and *r*_*pb*_ < .10) or not significantly correlated with the childhood and adulthood sex-typed variables (Table 3). Moreover, the direction and significance of our main variables did not change when controlling for the demographic variables.

**Table 2:** Descriptive statistics for demographic information by group

	Heterosexual Men (*n* = 270)	Gay Men (*n* = 199)	*Sao Praphet Song* (*n* = 166)	*Toms* (*n* = 174)	Lesbian Women (*n* = 56)	*Dees* (*n* = 149)	Heterosexual Women (*n* = 280)
Age *M* (*SD*)	25.21 (6.84)	22.85 (4.38)	24.83 (7.09)	25.83 (6.02)	23.48 (4.40)	24.22 (5.07)	27.91 (11.02)
*Level of Education*							
Secondary or lower	65.93%	71.36%	71.69%	47.13%	60.71%	55.70%	60.36%
Post-Secondary	34.07%	28.64%	28.31%	52.87%	39.29%	44.30%	39.64%
*Weekly Income (Thai Baht)*							
9,999 Baht or less	65.56%	74.37%	72.89%	57.47%	60.71%	56.38%	64.64%
10,000 Baht or more	34.44%	25.63%	27.11%	42.53%	39.29%	43.62%	35.36%

**Table 3:** Correlations between descriptive and sex-typed variables

Sex-typed variables	Age		Level of Education		Weekly Income	
	Pearson’s *r*	*p* value	Point biserial *r*_*pb*_	*p *value	Point biserial *r*_*pb*_	*p* value
CFTB	.038	.172	.002	.935	− . 001	.967
CMTB	.012	.667	.054	.052	.028	.310
CSAB	− **.080**	**.004**	− .009	.736	− .009	.755
FTOP	**.064**	**.021**	**.068**	**.014**	.035	.206
MTOP	**.099**	**< .001**	.045	.108	.028	.310
MF-Occ	− .036	.196	.010	.731	.001	.979

CFTB, childhood female-typical behavior; CMTB, childhood male-typical behavior; CSAB, childhood sex-atypical behavior; FTOP, female-typical occupational preferences; MTOP, male-typical occupational preferences; MF-Occ, male-versus-female-typical occupational preferences

Significant correlations are bolded

### Recalled Childhood Sex-Typed Behavior

**Fig. 1:**
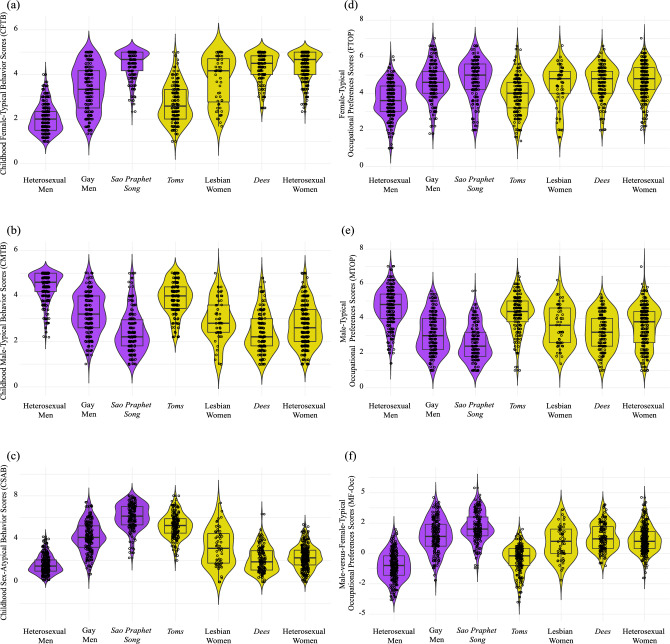
Recalled childhood sex-typed behaviors and adulthood occupational preferences by group. The panels show violin plots of (**a**) childhood female-typical behavior (CFTB) scores, **b** childhood male-typical behavior (CMTB) scores, **c** childhood sex-atypical behaviors (CSAB) scores, **d** female-typical occupational preferences (FTOP) scores, **e** male-typical occupational preferences scores, and **f** male-versus-female-typical occupational preferences (MF-Occ) scores by group. Values for CSAB scores (**c**) ranged from 0 to 8 with higher scores representing greater sex-atypical behavior. Values for MF-Occ scores (**f**) ranged from − 6 to 6 with positive scores representing greater female-typical behavior and negative scores representing greater male-typical behavior

Significant group differences were found in the average childhood female-typical and male-typical behavior scores and the composite childhood sex-atypical behavior score (Table 4 and Fig. 1). Below, we highlight significant heterosexual sex differences, significant sexual orientation group differences within sex, significant differences between same-sex attracted groups and the heterosexual group of the other sex, and their absolute Cohen’s *d* effect sizes. See Table 5 for a full list of all pairwise comparisons.

**Table 4:** Comparison of childhood sex-typed behavior scores and occupational preferences scores by group

	Heterosexual Men (*n* = 270)		Gay Men (*n* = 199)		*Sao Praphet Song* (*n* = 166)		*Toms* (*n* = 174)		Lesbian Women (*n* = 56)		*Dees* (*n* = 149)		Heterosexual Women (*n* = 280)		One-way ANOVA^a^			
	*M*	*SD*	*M*	*SD*	*M*	*SD*	*M*	*SD*	*M*	*SD*	*M*	*SD*	*M*	*SD*	*F*	*df*	*p*	*η*^2^
CFTB	2.01	0.63	3.39	1.01	4.48	0.61	2.70	0.84	3.74	1.08	4.34	0.64	4.41	0.61	496.65	399.89	< .001	0.63
CMTB	4.43	0.61	3.23	0.88	2.41	0.92	3.92	0.72	2.92	0.94	2.40	0.90	2.70	0.89	234.88	401.82	< .001	0.47
CSAB	1.57	0.82	4.16	1.40	6.08	1.17	5.22	1.10	3.18	1.74	2.06	1.17	2.29	1.06	501.80	396.53	< .001	0.67
FTOP	3.62	0.93	4.61	0.98	4.77	1.08	3.90	1.02	4.49	1.13	4.63	0.91	4.71	0.87	49.56	404.60	< .001	0.18
MTOP	4.66	1.03	3.12	1.13	2.57	1.01	4.33	1.03	3.56	1.14	3.26	1.04	3.56	1.16	96.72	409.91	< .001	0.29
MF-Occ	− 1.03	1.18	1.49	1.33	2.20	1.21	− 0.43	1.12	0.93	1.34	1.37	1.09	1.15	1.06	196.76	406.37	< .001	0.48

CFTB, childhood female-typical behavior; CMTB, childhood male-typical behavior; CSAB, childhood sex-atypical behavior; FTOP, female-typical occupational preferences; MTOP, male-typical occupational preferences; MF-Occ, male-versus-female-typical occupational preferences

Values for the MF-Occ scores ranged from − 6 to 6, with positive scores representing higher female-typical behavior and negative scores representing greater male-typical behavior. Values for CSAB scores ranged from 0 to 8, with higher scores representing greater sex-atypical behavior

^a^ Due to significant Levene’s tests for equality of variance (*p* < .05), all one-way ANOVAs were performed using Welch tests

**Table 5:** Post hoc pairwise comparisons of recalled childhood sex-typed behavior scores

Group Comparison	CFTB		CMTB		CSAB	
	*p*	Cohen’s *d* (95% CI)	*p*	Cohen’s *d* (95% CI)	*p*	Cohen’s *d* (95% CI)
Heterosexual Men—Gay Men	**< .001**	− **1.64 (**− **1.87, **− **1.43)**	**< .001**	**1.59 (1.34, 1.81)**	**< .001**	− **2.25 (**− **2.5, **− **2.00)**
Heterosexual Men—*Sao Praphet Song*	**< .001**	− **4.00 (**− **4.43, **− **3.54)**	**< .001**	**2.59 (2.25, 2.97)**	**< .001**	− **4.46 (**− **4.97, **− **3.96)**
Heterosexual Men—*Toms*	**< .001**	− **0.94 (**− **1.15, **− **0.74)**	**< .001**	**0.77 (0.58, 0.99)**	**< .001**	− **3.77 (**− **4.12, **− **3.37)**
Heterosexual Men—*Lesbian Women*	**< .001**	− **1.97 (**− **2.45, **− **1.48)**	**< .001**	**1.91 (1.55, 2.32)**	**< .001**	− **1.19 (**− **1.57, **− **0.84)**
Heterosexual Men—*Dees*	**< .001**	− **3.68 (**− **4.07, **− **3.20)**	**< .001**	**2.66 (2.26, 3.03)**	**< .001**	− **0.48 (**− **0.69, **− **0.29)**
Heterosexual Men—Heterosexual Women	**< .001**	− **3.91 (**− **4.31, **− **3.51)**	**< .001**	**2.27 (2.03, 2.53)**	**< .001**	− **0.75 (**− **0.92, **− **0.57)**
Gay Men—*Sao Praphet Song*	**< .001**	− **1.31 (**− **1.56, **− **1.09)**	**< .001**	**0.91 (0.69, 1.15)**	**< .001**	− **1.48 (**− **1.75, **− **1.23)**
Gay Men—*Toms*	**< .001**	**0.74 (0.52, 0.96)**	**< .001**	− **0.86 (**− **1.04, **− **0.62)**	**< .001**	− **0.84 (**− **1.06, **− **0.63)**
Gay Men—Lesbian Women	.316	− 0.34 (− 0.66, − 0.02)	.317	0.34 (0.02, 0.63)	**.004**	**0.62 (0.28, 0.93)**
Gay Men—*Dees*	**< .001**	− **1.12 (**− **1.35, **− **0.89)**	**< .001**	**0.93 (0.70, 1.17)**	**< .001**	**1.63 (1.35, 1.89)**
Gay Men—Heterosexual Women	**< .001**	− **1.23 (**− **1.43, **− **1.03)**	**< .001**	**0.59 (0.41, 0.79)**	**< .001**	**1.50 (1.29, 1.74)**
*Sao Praphet Song*—*Toms*	**< .001**	**2.42 (2.06, 2.78)**	**< .001**	− **1.83 (**− **2.15, **− **1.53)**	**< .001**	**0.76 (0.51, 0.97)**
*Sao Praphet Song*—Lesbian Women	**< .001**	**0.85 (0.50, 1.17)**	**.010**	− **0.55 (**− **0.90, **− **0.19)**	**< .001**	**1.95 (1.51, 2.39)**
*Sao Praphet Song*—*Dees*	.371	0.23 (0.03, 0.45)	1.00	0.01 (− 0.21, 0.23)	**< .001**	**3.44 (2.97, 3.84)**
*Sao Praphet Song*—Heterosexual Women	.902	0.12 (− 0.07, 0.31)	**.019**	− **0.32 (**− **0.52, **− **0.09)**	**< .001**	**3.39 (3.01, 3.75)**
*Toms*—Lesbian Women	**< .001**	− **1.07 (**− **1.42, **− **0.70)**	**< .001**	**1.19 (0.84, 1.57)**	**< .001**	**1.40 (0.97, 1.79)**
*Toms*—*Dees*	**< .001**	− **2.18 (**− **2.51, **− **1.86)**	**< .001**	**1.88 (1.59, 2.18)**	**< .001**	**2.79 (2.39, 3.11)**
*Toms*—Heterosexual Women	**< .001**	− **2.33 (**− **2.64, **− **2.00)**	**< .001**	**1.51 (1.27, 1.75)**	**< .001**	**2.71 (2.42, 2.98)**
Lesbian Women—*Dees*	**.004**	− **0.68 (**− **1.02, **− **0.34)**	**.009**	**0.57 (0.24, 0.92)**	**< .001**	**0.76 (0.42, 1.09)**
Lesbian Women—Heterosexual Women	**< .001**	− **0.78 (**− **1.09, **− **0.47)**	.667	0.24 (− 0.07, 0.53)	**.007**	**0.62 (0.28, 0.97)**
*Dees*—Heterosexual Women	.894	− 0.12 (− 0.32, 0.09)	**.016**	− **0.34 (**− **0.55, **− **0.12)**	.431	− 0.20 (− 0.41, 0.00)

CFTB, childhood female-typical behavior; CMTB, childhood male-typical behavior; CSAB, childhood sex-atypical behavior

All pairwise comparisons were conducted using the Games-Howell procedure due to differences in sample size and significant Levene’s tests for equality of variance (*p* < .05). Significant pairwise comparisons are bolded

With respect to childhood female-typical behaviors, heterosexual women recalled significantly higher levels than heterosexual men (*d* = 3.91). Among females, *toms* recalled significantly less CFTB than heterosexual women (*d* = 2.33), *dees* (*d* = 2.18) and lesbian women (*d* = 1.07), and lesbian women recalled significantly less CFTB than heterosexual women (*d* = 0.78) and *dees* (*d* = 0.68). Among males, heterosexual men recalled significantly less CFTB than gay men (*d* = 1.64) and *sao praphet song* (*d* = 4.00), and *sao praphet song* recalled significantly more CFTB than gay men (*d* = 1.31). In addition, heterosexual women recalled significantly more CFTB than gay men (*d* = 1.23), and heterosexual men recalled significantly less CFTB than *dees* (*d* = 3.68), lesbian women (*d* = 1.97), and *toms* (*d* = 0.94).

With respect to childhood male-typical behaviors, heterosexual men recalled significantly higher levels than heterosexual women (*d* = 2.27). Among females, *toms* recalled significantly more CMTB than heterosexual women (*d* = 1.51), *dees* (*d* = 1.88), and lesbian woman (*d* = 1.19), and *dees* recalled less CMTB than heterosexual women (*d* = 0.34) and lesbian women (*d* = 0.57). Among males, heterosexual men recalled significantly more CMTB than gay men (*d* = 1.59) and *sao praphet song* (*d* = 2.59), and gay men recalled significantly more CMTB than *sao praphet song* (*d* = 0.91). In addition, heterosexual women recalled significantly less CMTB than gay men (*d* = 0.59) and *sao praphet song* (*d* = 0.32), and heterosexual men recalled significantly more CMTB than *dees* (*d* = 2.66), lesbian women (*d* = 1.91), and *toms* (*d* = 0.77).

Finally, with respect to childhood sex-atypical behavior composite scores, heterosexual women recalled significantly higher levels than heterosexual men (*d* = 0.75). Among females, *toms* recalled significantly more CSAB than heterosexual women (*d* = 2.71), *dees* (*d* = 2.79), lesbian women (*d* = 1.40), and lesbian women recalled significantly more CSAB than heterosexual women (*d* = 0.62) and *dees* (*d* = 0.76). Among males, *sao praphet song* recalled significantly more CSAB than heterosexual men (*d* = 4.46) and gay men (*d* = 1.48), and gay men recalled significantly more CSAB than heterosexual men (*d* = 2.25). In addition, heterosexual women recalled significantly less CSAB than gay men (*d* = 1.50) and *sao praphet song* (*d* = 3.39), and heterosexual men recalled significantly less CSAB than *dees* (*d* = 0.48), lesbian women (*d* = 1.19), and *toms* (*d* = 3.77).

### Adult Occupational Preferences

Significant group differences were found in the average female-typical and male-typical occupational preferences score and the composite male-versus female-typical occupational preference score (Table 4 and Fig. 1). Below, we highlight significant heterosexual sex differences, significant sexual orientation group differences within sex, significant differences between same-sex attracted groups and the heterosexual group of the other sex, and their absolute Cohen’s *d* effect sizes. See Table 6 for a full list of all pairwise comparisons.

**Table 6:** Post hoc pairwise comparisons of occupational preferences scores

Group Comparison	FTOP		MTOP		MF-Occ	
	*p*	Cohen’s *d* (95% CI)	*p*	Cohen’s *d* (95% CI)	*p*	Cohen’s *d* (95% CI)
Heterosexual Men—Gay Men	**< .001**	− **1.03 (**− **1.23, **− **0.82)**	**< .001**	**1.42 (1.21, 1.67)**	**< .001**	− **2.01 (**− **2.22, **− **1.78)**
Heterosexual Men—*Sao Praphet Song*	**< .001**	− **1.13 (**− **1.37, **− **0.90)**	**< .001**	**2.04 (1.79, 2.31)**	**< .001**	− **2.70 (**− **2.96, **− **2.42)**
Heterosexual Men—*Toms*	.070	− 0.28 (− 0.46, − 0.08)	**.019**	**0.32 (0.13, 0.51)**	**< .001**	− **0.52 (**− **0.72, **− **0.34)**
Heterosexual Men—*Lesbian Women*	**< .001**	− **0.84 (**− **1.22, **− **0.48)**	**< .001**	**1.00 (0.67, 1.31)**	**< .001**	− **1.55 (**− **1.87, **− **1.19)**
Heterosexual Men—*Dees*	**< .001**	− **1.09 (**− **1.32, **− **0.88)**	**< .001**	**1.34 (1.12, 1.57)**	**< .001**	− **2.11 (**− **2.33, **− **1.86)**
Heterosexual Men—Heterosexual Women	**< .001**	− **1.21 (**− **1.41, **− **1.02)**	**< .001**	**0.99 (0.82, 1.18)**	**< .001**	− **1.95 (**− **2.14, **− **1.75)**
Gay Men—*Sao Praphet Song*	.758	− 0.16 (− 0.36, 0.04)	**< .001**	**0.51 (0.29, 0.71)**	**< .001**	− **0.56 (**− **0.76, **− **0.32)**
Gay Men—*Toms*	**< .001**	**0.71 (0.48, 0.92)**	**< .001**	− **1.12 (**− **1.36, **− **0.87)**	**< .001**	**1.56 (1.34, 1.77)**
Gay Men—Lesbian Women	.992	0.11 (− 0.21, 0.42)	.139	− 0.39 (− 0.70, − 0.11)	.089	0.42 (0.15, 0.74)
Gay Men—*Dees*	1.00	− 0.03 (− 0.27, 0.18)	.867	− 0.14 (− 0.37, 0.08)	.964	0.10 (− 0.11, 0.31)
Gay Men—Heterosexual Women	.879	− 0.12 (− 0.29, 0.07)	**< .001**	− **0.39 (**− **0.58, **− **0.20)**	.051	0.28 (0.10, 0.46)
*Sao Praphet Song*—*Toms*	**< .001**	**0.83 (0.6, 1.07)**	**< .001**	− **1.72 (**− **2.02, **− **1.44)**	**< .001**	**2.25 (2.00, 2.46)**
*Sao Praphet Song*—Lesbian Women	.676	0.25 (− 0.02, 0.59)	**< .001**	− **0.92 (**− **1.25, **− **0.62)**	**< .001**	**1.00 (0.67, 1.34)**
*Sao Praphet Song*—*Dees*	.888	0.14 (− 0.10, 0.36)	**< .001**	− **0.68 (**− **0.91, **− **0.44)**	**< .001**	**0.72 (0.51, 0.96)**
*Sao Praphet Song*—Heterosexual Women	.998	0.05 (− 0.13, 0.28)	**< .001**	− **0.91 (**− **1.14, **− **0.72)**	**< .001**	**0.92 (0.69, 1.11)**
*Toms*—Lesbian Women	**.012**	− **0.55 (**− **0.91, **− **0.20)**	**< .001**	**0.70 (0.40, 1.06)**	**< .001**	− **1.10 (**− **1.42, **− **0.76)**
*Toms*—*Dees*	**< .001**	− **0.76 (**− **0.96, **− **0.51)**	**< .001**	**1.02 (0.76, 1.26)**	**< .001**	− **1.62 (**− **1.83, **− **1.41)**
*Toms*—Heterosexual Women	**< .001**	− **0.86 (**− **1.07, **− **0.65)**	**< .001**	**0.70 (0.49, 0.92)**	**< .001**	− **1.45 (**− **1.62, **− **1.25)**
Lesbian Women—*Dees*	.980	− 0.14 (− 0.47, 0.19)	.608	0.27 (− 0.04, 0.61)	.305	− 0.36 (− 0.70, − 0.06)
Lesbian Women—Heterosexual Women	.796	− 0.22 (− 0.52, 0.08)	1.00	0.00 (− 0.28, 0.29)	.892	− 0.19 (− 0.49, 0.15)
*Dees*—Heterosexual Women	.970	− 0.09 (− 0.30, 0.09)	.104	− 0.27 (− 0.47, − 0.07)	.454	0.20 (0.00, 0.41)

FTOP, female-typical occupational preferences; MTOP, male-typical occupational preferences; MF-Occ, male-versus-female-typical occupational preferences

All pairwise comparisons were conducted using the Games-Howell procedure due to differences in sample size and significant Levene’s tests for equality of variance (*p* < .05). Significant pairwise comparisons are bolded

With respect to female-typical occupational preferences, heterosexual women reported significantly higher levels than heterosexual men (*d* = 1.13). Among females, *toms* reported significantly lower FTOP than heterosexual women (*d* = 0.86), *dees* (*d* = 0.76), and lesbian women (*d* = 0.55). Among males, heterosexual men reported significantly lower FTOP than gay men (*d* = 1.03) and *sao praphet song* (*d* = 1.13). In addition, heterosexual men reported significantly lower FTOP than *dees* (*d* = 1.09) and lesbian women (*d* = 0.84).

With respect to male-typical occupational preferences, heterosexual men reported significantly higher levels than heterosexual women (*d* = 0.99). Among females, *toms* reported significantly greater MTOP than heterosexual women (*d* = 0.70), *dees* (*d* = 1.02), and lesbian women (*d* = 0.70). Among males, heterosexual men reported significantly greater MTOP than gay men (*d* = 1.42) and *sao praphet song* (*d* = 2.04), and gay men had significantly greater MTOP than *sao praphet song* (*d* = 0.51). In addition, heterosexual women reported significantly lower MTOP than gay men (*d* = 0.39) and *sao praphet song* (*d* = 0.91), and heterosexual men reported significantly greater MTOP than *dees* (*d* = 1.34), lesbian women (*d* = 1.00), and *toms* (*d* = 0.32).

Lastly, with respect to male-versus female-typical occupational preferences, heterosexual men had significantly lower scores than heterosexual women (*d* = 1.95), indicating lower female-typical and greater male-typical occupational preferences. Among females, *toms* had significantly lower MF-Occ scores than heterosexual women (*d* = 1.45), *dees* (*d* = 1.62), and lesbian women (*d* = 1.10). Among males, heterosexual men had significantly lower MF-Occ scores than gay men (*d* = 2.01) and *sao praphet song* (*d* = 2.70), and *sao praphet song* had significantly greater MF-Occ scores than gay men (*d* = 0.56). In addition, heterosexual women had significantly lower MF-Occ scores than *sao praphet song* (*d* = 0.92), and heterosexual men had significantly lower MF-Occ scores than *dees* (*d* = 2.11), lesbian women (*d* = 1.55), and *toms* (*d* = 0.52).

### Associations Between Recalled Childhood Sex-Atypical Behavior and Adult Occupational Preferences

Significant correlations were found between CSAB and MF-Occ among heterosexual men (*r* = .18, *p* = .004), gay men (*r* = .43, *p* < .001), *sao praphet song* (*r* = .32, *p* < .001), and all male participants combined (*r* = .74, *p* < .001). Significant correlations were also found between CSAB and MF-Occ among *toms* (*r* = − .20, *p* = .008), lesbian women (*r* = − .31, *p* = .020), *dees* (*r* = − .33, *p* < .001), and all female participants combined (*r* = − .52, *p* < .001), but not for heterosexual women (*r* = − .09, *p* = .122). Comparing the absolute strengths of these associations between all males and all females using Fisher’ *r* to *z* transformation revealed that the correlation between CSAB and MF-Occ was significantly stronger among males than females (*z* = 6.73, *p* < .001, Cohen’s *q* = 0.38).

Model 1 of the linear regression analysis in Table 7 revealed that among males, CSAB was a significant predictor of adult MF-Occ (*β* = 0.74,* p* < .001), accounting for 55% of the variance. Model 2 revealed that among males, CSAB significantly predicted adult MF-Occ scores independent of sexual orientation (*β* = 0.40, *p* < .001). The significant increase in the variance explained between Models 1 and 2 (∆*R*^2^ = .067, *p* < .001) suggests that male sexual orientation predicts MF-Occ scores even when controlling CSAB. Indeed, Model 2 revealed that compared with heterosexual men, being in the gay men (*β* = 0.40, *p* < .001) or *sao praphet song* (*β* = 0.38, *p* < .001) group was an independent predictor of higher MF-Occ scores. Altogether, the predictors in Model 2 accounted for 62% of the variance in MF-Occ scores.

Model 1 of the linear regression analysis in Table 8 revealed that among females, CSAB was a significant predictor of adult MF-Occ (*β* = − 0.52,* p* < .001), accounting for 27% of the variance. Model 2 revealed that among females, CSAB significantly predicted adult MF-Occ scores independent of sexual orientation (*β* = − 0.26, *p* < .001). The significant increase in the variance explained between Model 1 and 2 (∆*R*^2^ = .058, *p* < .001) suggests that female sexual orientation predicts MF-Occ scores even when controlling for CSAB. However, Model 2 revealed that compared with heterosexual women, being in the *tom* (*β* = − 0.34, *p* < .001) group was an independent predictor of lower MF-Occ scores whereas being in the group of *dees* (*β* = 0.05, *p* = .128) or lesbian women (*β* = − 0.01, *p* = .748) was not. Altogether, the predictors in Model 2 accounted for 32% of the variance in MF-Occ scores.

**Table 7:** Linear regression predicting male- versus female-typical occupational preferences scores based on gender and childhood sex-atypical behavior scores for male groups

		Male-versus-female-typical occupational preferences					
		*B*	95% CI	SE	*β*	*t*	*p*
*Model*	*Predictor*						
1	Childhood sex-atypical behavior	0.65	0.60, 0.69	0.02	0.74	27.80	**< **.001
2	Childhood sex-atypical behavior	0.35	0.27, 0.43	0.04	0.40	8.47	**< **.001
	Heterosexual Men vs Gay Men	1.61	1.31, 1.91	0.15	0.40	10.51	**< **.001
	Heterosexual Men vs *Sao Praphet Song*	1.64	1.21, 2.08	7.47	0.38	7.47	**< **.001

Model 1:* R*^2^ = .55; Adjusted *R*^2^ = .549; *F*(1, 633) = 773, *p* < .001

Model 2: *R*^2^ = .617; Adjusted *R*^2^ = .615; *F* (3, 631) = 338.79, *p* < .001

∆*R*^2^ = .067, *F* (2, 631) = 55.34, *p* < .001

For both dummy variables, heterosexual men were coded as 0. In Heterosexual Men vs Gay Men, *sao praphet song* were coded as 0 and gay men as 1. In Heterosexual Men vs *Sao Praphet Song*, gay men were coded as 0 and *sao praphet song* as 1

## Discussion

The present study examined whether there are differences in childhood and adulthood sex-typed behaviors between and within male and female groups of varying gender/sexual orientation identities in Thailand. This unique diversity of gender expression among same-sex attracted males *and* females in Chiang Mai, Thailand, provided a valuable opportunity to assess the extent to which sex, sexual orientation, and gender-role presentation are related to sex-typed behaviors and interests—something that has been rarely explored in previous studies. Further, this is only the second study to quantitatively assess the adulthood occupational preferences of transgender/gender-diverse (TGD) females.

Consistent with our first prediction and previous research (e.g., Lippa, 2008a, 2010), Thai heterosexual men and women demonstrated large sex differences in childhood (CFTB: *d* = 3.22; CMTB: *d* = 2.10) and adulthood (FTOP: *d* = 1.13; MTOP: *d* = 1.01; MF-Occ: *d* = 1.13) sex-typed behaviors and interests. Furthermore, heterosexual women also recalled significantly more sex-atypical behaviors in childhood compared with heterosexual men. This result is consistent with previous studies in Canada (VanderLaan et al., 2011), the USA (Lippa, 2008b), and the Istmo region of Oaxaca, Mexico (Gómez Jiménez et al., 2021), and suggests that girls across these cultures face less social pressure to behave in a sex-typical manner than boys. Consistent with this possibility, research conducted in Canada (e.g., Nabbijohn et al., 2020), the USA (e.g., Coyle et al., 2016), the Netherlands (e.g., Wallien & Cohen-Kettenis, 2008), and Hong Kong (e.g., Kwan et al., 2020) indicates that the expression of sex-atypical behaviors in boys is typically viewed more negatively than in girls. However, similar research in Thai children reported no such pattern (Wang et al., 2022), making it less clear whether social pressure to conform to gender norms influenced the heterosexual sex differences in degree of sex-atypicality observed here.

With respect to male sexual orientation differences, the present study found that both gay men and *sao praphet song* exhibited more female-typical and less male-typical—and, thus, more sex-atypical—childhood play behaviors and adulthood occupational preferences than heterosexual men. Moreover, gay men were intermediate between heterosexual men and *sao praphet song* for all variables except for FTOP—for which *sao praphet song* and gay men showed more similar scores. These findings provide further evidence to suggest that having elevated sex-atypical childhood and adulthood behaviors and interests are cross-culturally universal aspects of male same-sex sexuality, regardless of gender-role presentation (i.e., masculine- or feminine-presenting).

While the male sexual orientation differences in sex-typed behaviors were largely consistent with our predictions, the manner by which the androphilic male groups differed from heterosexual women varied between the childhood and adulthood variables. With respect to childhood sex-typed behaviors, gay men scored in between heterosexual men and women for both male- and female-typical behaviors, whereas *sao praphet song* were as feminine as (but less masculine than) heterosexual women. These results are consistent with prior research demonstrating that cisgender androphilic males exhibit a pattern of childhood sex-typed behavior that is female-shifted (e.g., Bailey & Zucker, 1995; Gómez Jiménez et al., 2021; Sadr-Bazzaz et al., 2024a; Singh et al., 2021; VanderLaan et al., 2011, 2015; Wallien & Cohen-Kettenis, 2008), whereas TGD androphilic males exhibit a pattern that either mirrors or exaggerates the pattern observed among heterosexual women (e.g., Gómez Jiménez et al., 2021; Roshan et al., 2019; Sadr-Bazzaz et al., 2024a; Semenyna & Vasey, 2016; Singh et al., 2021; VanderLaan et al., 2017; Wallien & Cohen-Kettenis, 2008).

With respect to adulthood occupational preferences, however, both *sao praphet song* and gay men were as female-typical as, but less male-typical than, heterosexual women. Thus, the occupational preferences exhibited by Thai gay men in the present study were not consistent with those found among gay men in Euro-American cultures, who tend to be intermediate between heterosexual men and women (e.g., Ellis et al., 2012; Lippa, 2005a, 2008a, 2008b, 2020; VanderLaan et al., 2016; for similar evidence in Chinese gay men, see Zheng et al., 2011). Nevertheless, like the present study, Gómez Jiménez et al. (2021) found that heterosexual women had greater male-typical occupational preferences than both masculine- and feminine-presenting androphilic males. The authors speculated that this could be due to a greater aversion toward male-typical occupations among masculine- and feminine-presenting androphilic males in the Istmo region of Oaxaca, Mexico, who tend to identify or be identified as members of a non-binary gender locally known as *muxe* as early as 3 years of age (Chiñas, 1995; Miano Borruso, 2002). In other words, identification as a member of a non-binary gender group that is markedly feminine might reduce the internal/social pressures to behave masculine among *muxes* relative to gay men in the West.

Similarly, it is possible that gay men in Thailand, who are also often considered to be *pheet thii saam* (i.e., a “third sex/gender”; Sinnott, 2004), face less internal and external pressure to be masculine relative to gay men in the West. This might help explain why Thai gay men in the present study exhibited patterns of adulthood occupational preferences that mirrored those of heterosexual women. On the other hand, prejudice against the more male-typical, cisgender expression of male androphilia in Thailand has been previously described (Jackson, 1999). Thus, it is possible that the present study did not sample enough androphilic males on the male-typical end of the spectrum or that they identified as heterosexual men because they did not feel comfortable disclosing their same-sex attraction and behavior. Future studies could test these possibilities by comparing levels of internal and external homo-/transphobia across cultures and assessing the extent to which this might influence investigations of sex-typed behaviors and interests among and between cisgender and TGD androphilic males.

With respect to female group differences, the present study found that *toms* reported having more male-typical and less female-typical—and, thus, more sex-atypical—childhood play behaviors and adulthood occupational preferences than heterosexual women, *dees*, and lesbian women. In contrast, lesbian women reported greater sex-atypical behaviors/interests in childhood, but not adulthood, than heterosexual women and *dees*. Specifically, lesbian women recalled lower female-typical childhood behaviors than heterosexual women and *dees*, and greater male-typical childhood behaviors than *dees*. Finally, *dees* only differed from heterosexual women by recalling lower male-typical behaviors in childhood.

The findings for the female sexual orientation differences suggest that the expression of sex-atypical behaviors and interests might not be a strong correlate of female *gynephilia*. This is because, unlike our findings from our same-sex attracted male samples, only same-sex attracted females with a masculine gender-role presentation (i.e., *toms*) differed from heterosexual women with respect to *both* childhood and adulthood sex-typed behaviors and interests. In contrast, same-sex attracted females with a feminine gender-presentation were either sex-atypical only in childhood (i.e., lesbian women) or not sex-atypical at all (i.e., *dees*). It is possible, then, that among females, gender-role presentation/gender identity is a stronger correlate than sexual orientation of sex-typed behaviors and interests.

When compared with both heterosexual men and women, the childhood and adulthood sex-typed behaviors and interests for *toms* were largely intermediate. In contrast, Sadr-Bazzaz et al., (2024a, 2024b) found that Iranian transgender gynephilic females had a pattern of sex-typed behaviors that either mirrored (in childhood) or exaggerated (in adulthood) those observed among heterosexual individuals of the other sex, similar to the pattern observed among *sao praphet song* in the present study. It is worth noting that, in addition to *toms*, there also exist TGD females in Thailand who identify as transgender men. We did not sample enough transgender men for quantitative analysis in the present study, but it is not unreasonable to postulate that they might evince a pattern of sex-typed behaviors that mirrors those of heterosexual men. As such, further studies are needed to understand more fully the extent to which TGD females differ from heterosexual men and women with respect to their childhood sex-typed behaviors and adulthood occupational preferences.

Contrary to our predictions, the childhood and adulthood sex-typed behaviors and interests of Thai lesbian women were broadly similar to those of heterosexual women, with the exception of CFTB for which lesbian women were intermediate compared with heterosexual men and women. These results are inconsistent with studies from cisgender gynephilic females in other cultures, who typically exhibit sex-typed behaviors and interests that are shifted in the male-direction (e.g., Bailey & Zucker, 1995; Ellis et al., 2012; Lippa, 2005a, 2008a, 2008b, 2020; Sadr-Bazzaz et al., 2024a, 2024b; Zheng et al., 2011). It is worth noting, however, that the sample size of lesbian women (*n* = 56) in the present study was relatively small. While it is difficult to ascertain whether identification as lesbian is as common as identification as *tom* or *dee* in Thailand, some scholarly writings on Thai sexual and gender diversity have highlighted how the category “lesbian” has gained popularity in Thailand more recently (e.g., Jackson, 2000; Miedema et al., 2022). This might explain the lower number of lesbians we were able to recruit into the study. Nevertheless, it is possible that the smaller sample size of lesbian women might not have provided sufficient power to detect significant differences between lesbian and heterosexual women in CMTB, MTOP, FTOP, and MF-Occ.

A power analysis can shed light on this possibility. Previous meta-analyses based on Euro-American samples have found that the differences between heterosexual and lesbian women in recalled childhood sex-typed behaviors (*d* = 0.96; Bailey & Zucker, 1995) and adulthood occupational preferences (*d* = 1.46; Lippa, 2005a) tend to be rather large. Power analyses based on these effect sizes indicated that the present study would only require a total sample size of *N* = 30 to detect similar differences between heterosexual and lesbian women.^5^ As such, it seems unlikely that statistical power issues were responsible for the lack of general differences between lesbian and heterosexual women observed here.

An alternate explanation for the cross-cultural inconsistency in lesbian women’s sex-typed behaviors and interests relative to those of heterosexual women relies on differences in the identity categories afforded to gynephilic females in Thailand versus other cultures. In Euro-American cultures, subgroups of masculine and feminine female gynephiles may be similarly likely to identify as lesbian (although for discussion of possible changes to this trend in recent years, see Leinung & Joseph, 2020). In Iran, where cisgender gynephilic females have been found to exhibit either male-shifted or male-typical patterns of sex-typed behaviors, only those with significant levels of distress or dissatisfaction with their bodies are legally and medically allowed to live as another gender (Sadr-Bazzaz et al., 2024a, 2024b). This would make it less likely (and more difficult) for masculine gynephilic females in Iran to identify as transgender men. By contrast, given the visibility and relatively greater acceptance of gender diversity in Thailand (e.g., Sinnott, 2004), female gynephiles who are attracted to cisgender women and more markedly masculine may more readily identify with the social category of *tom*, whereas those who are less masculine may more readily identify with the social category of lesbian. As has been emphasized elsewhere, it is important to stress that identification with the social category of *tom* holds significant and culturally specific meaning such that it would be erroneous to draw an equivalency between *toms* and lesbians (Sinnott, 2004). Consistent with this idea, while both lesbian women and *toms* were sexually attracted to cisgender women (Table 1), *toms* reported being more masculine than lesbian women in both childhood and adulthood (Tables 5, 6). It is possible, then, that lesbian women in Thailand are more feminine as a group relative to cisgender gynephilic females in Iran and Euro-American cultures, which would explain the inconsistencies in results across cultural contexts.

If the expression of sex-atypical behaviors and interests were a cross-culturally universal correlate of female gynephilia, we would have expected to see more male-typical childhood behaviors and occupational preferences among *dees* compared with heterosexual women; however, such was not the case in the present study. That said, it is worth noting that unlike lesbian women and *toms*, *dees* are specifically attracted to transmasculine *toms* rather than cisgender females (i.e., women; see Table 1). Thus, although it would be accurate to describe *dees* as gynephilic given that they are sexually attracted to individuals assigned female at birth, the fact that they are particularly attracted to a subset of females who are masculine might make them different as a group from lesbian women and *toms*, who are mainly attracted to feminine females. To our knowledge, this is the first study to assess the sex-typed behaviors and interests of a group of females who are sexually attracted to transmasculine individuals. Thus, further research is needed to better understand the extent to which the expression of sex-atypical behaviors and interests among females correlates with sexual attraction to feminine females, females in general (regardless of gender expression), and/or masculine gender norms/roles adherence. Here, the sex-typed behaviors and interests of *dees* seemed to most align with those of the other female groups who reported attraction to masculine individuals (i.e., heterosexual women), suggesting that attraction to masculine- vs. feminine-presenting individuals may be an important dimension.

While the present study does not directly assess the biodevelopmental origins of same-sex sexuality and sex-atypicality, the findings from our group comparisons can provide some insights to this end. As discussed in the Introduction, one possible explanation for same-sex sexuality posits that neurohormonal factors have additive effects during pre-/peri-natal development, such that some level of exposure/receptivity to sex-atypical hormones leads to same-sex attraction and some sex-atypical behaviors and interests, whereas higher levels lead to both same-sex attraction and greater sex-atypicality (reviewed in VanderLaan et al., 2023). Consistent with this idea, *sao praphet song* displayed levels of sex-typed behaviors and interests that largely mirrored heterosexual women’s, whereas those of gay men were intermediate between those of heterosexual men and *sao praphet song*. Other Thai studies on handedness (Skorska et al., 2020), visuospatial abilities (Thurston et al., 2021), and body measurements (e.g., leg length; Skorska et al., 2021)—all of which are thought to be influenced by sex hormones during sensitive periods of development—have also found patterns consistent with reduced androgen action on the development of *sao praphet song* in particular, and to a lesser extent on the development of gay men. Thus, the overall evidence from these studies is consistent with the idea that male androphilia in Thailand is influenced by neurohormonal factors that operate in a dosage-dependent manner.

Among females, the pattern is not as clear. Male-typical behaviors and interests were not found consistently among all female gynephiles in the present study. Only *toms* exhibited a male-shifted pattern for childhood and adulthood sex-typed behaviors and interests, and this effect was subdued relative to the pattern found among *sao praphet song*, who mirrored heterosexual women. Similarly, a previous study on body measurements in Thailand did not find consistent sexual orientation differences but instead found more male-typical patterns of weight and leg length among *toms* and lesbian when compared with heterosexual women and *dees* (Skorska et al., 2021). With respect to cognitive abilities, although Thai lesbian (and bisexual) women exhibited more male-typical visuospatial abilities than *dees*, the groups of *toms* and heterosexual women did not differ from all other female groups (Thurston et al., 2021). Overall, support for a neurohormonal explanation of same-sex sexuality and sex-atypicality in Thailand does not appear to be as consistent for females as it is for males.

VanderLaan et al. (2023) discussed the possibility of multiple biodevelopmental pathways (e.g., hormonal, genetic, immunological) toward same-sex sexuality, with varying influences on sex-typed behaviors. Thus, it is possible that among females, the mutual expression of same-sex attraction and sex-atypicality is not explained by a single biological factor (e.g., androgens). Instead, it could be that there is a distinct biodevelopmental pathway that leads to the expression of same-sex sexuality without (or reduced) sex-atypicality and another that leads to both same-sex sexuality and sex-atypicality. This would partially explain the inconsistency in female sexual orientation effects found here and in previous Thai studies (Skorska et al., 2021; Thurston et al., 2021).

With respect to the continuity between childhood and adulthood measures, the present study found that recalling sex-atypical behaviors in childhood was associated with having sex-atypical interests in adulthood among both males and females. Thus, similar to prior Euro-American (Lippa, 2008b; VanderLaan et al., 2016) and non-Euro-American (Gómez Jiménez et al., 2021; Sadr-Bazzaz et al., 2024b; Semenyna & Vasey, 2016) studies, the present study suggests there is a developmental continuity between childhood and adulthood sex-atypicality regardless of sex. These findings enhance our understanding of the development of gender diversity because they demonstrate that across cultures, individuals who are sex-atypical in childhood are likely to be sex-atypical in adulthood. This developmental continuity has also been found in prospective studies in Euro-American cultures (e.g., Green, 1987; Li et al., 2017; Singh et al., 2021; Steensma et al., 2013; Wallien & Cohen-Kettenis, 2008; Zucker & Bradley, 1995) but has only been documented using retrospective measures in non-Western studies. Thus, future studies could further corroborate the cross-cultural consistency in the developmental continuity of sex-atypicality by using longitudinal measures in non-Euro-American cultures.

While the correlation between childhood and adulthood sex-atypicality was independent of sex, the strength of this association appeared to be stronger for males (*r* = .74) than for females (*r* = − .52). There are various reasons why females who display sex-typical behaviors and interests might still prefer male-typical occupations over female-typical ones. For example, using data from the National Statistics Office in Thailand from 2008 to 2017, Paweenawat and Liao (2024) found that both women and men working in male-dominated occupations earned more than women and men working in female-dominated occupations, respectively. This suggest that sex-typical females have more to gain—at least in terms of income—from seeking male-typical occupations than sex-typical males have from seeking female-typical occupations. This could potentially explain why the correlation between sex-atypicality in childhood and sex-atypicality in adulthood occupational preferences was lower among females when compared with males. Future research could investigate this possibility by qualitatively exploring the reasons behind why Thai participants prefer particular types of occupations.

Further exploring the association between childhood and adulthood sex-atypicality, regression analyses revealed that among females, identifying as a *tom* as opposed to a heterosexual woman was associated with having sex-atypical occupational preferences in adulthood even when controlling for CSAB scores, whereas the same was not true for those who identified as a *dee* or lesbian woman. Therefore, it is possible that the significant increase in the variance explained between Models 1 and 2 in Table 8 was primarily driven by the gender differences between heterosexual women and *toms* rather than any female sexual orientation differences. In contrast, among males, identifying as a gay man or a *sao praphet song* as opposed to a heterosexual man were both independently associated with greater sex-atypical occupational preferences in adulthood when controlling for CSAB. Thus, once again, our results suggest that among females, gender-role presentation/gender identity play a stronger role than sexual orientation in predicting sex-typed behaviors and interests.

**Table 8:** Linear regression predicting male- versus female-typical occupational preferences scores based on gender and childhood sex-atypical behavior scores for female groups

		Male-versus-female-typical occupational preferences					
		*B*	95% CI	SE	*β*	*t*	*p*
*Model*	*Predictor*						
1	Childhood sex-atypical behavior	− 0.39	− 0.44, − 0.34	0.03	− 0.52	− 15.64	**< **.001
2	Childhood sex-atypical behavior	− 0.20	− 0.27, − 0.12	0.04	− 0.26	− 5.36	**< **.001
	Heterosexual Women vs *Dees*	0.17	− 0.05, 0.39	0.11	0.05	1.53	.128
	Heterosexual Women vs Lesbian Women	− 0.05	− 0.37, 0.27	0.16	− 0.01	− 0.32	.748
	Heterosexual Women vs* Toms*	− 1.01	− 1.30, − 0.71	0.15	− 0.34	− 6.73	**< **.001

Model 1:* R*^2^ = .271; Adjusted *R*^2^ = .270; *F*(1, 657) = 224.64, *p* < .001

Model 2: *R*^2^ = .329; Adjusted *R*^2^ = .325; *F* (3, 654) = 80.13, *p* < .001

∆*R*^2^ = .058, *F*(3, 654) = 18.70, *p* < .001

For the three dummy variables, heterosexual women were coded as 0. In Heterosexual Women vs *Dees*, lesbian women and *toms* were coded as 0 and *dees* as 1. In Heterosexual Women vs Lesbian Women, *dees* and *toms* were coded as 0 and lesbian women as 1. In Heterosexual Women vs *Toms, dees* and lesbian women were coded as 0 and *toms* as 1

### Limitations

The present study had a few limitations that are worth noting. First, network sampling procedures were used to find and recruit a convenience sample of participants, which may have produced an unrepresentative sample. Attempts were made to avoid such bias by interviewing participants from various towns and villages within and surrounding Chiang Mai. Nonetheless, future research in this area could be strengthened by utilizing probability sampling to obtain more representative samples. Second, participants’ sex-typed behaviors in childhood were assessed using retrospective measures, which could be prone to selective recall bias and memory distortion (e.g., Fausto-Sterling, 2014; Gottschalk, 2003; Maughan et al., 1997; Ross, 1980). While the expression of childhood sex-atypical behaviors among same-sex attracted individuals has also been found using prospective measures (e.g., Green, 1987; Li et al., 2017; Singh et al., 2021; Steensma et al., 2013; Wallien & Cohen-Kettenis, 2008; Zucker & Bradley, 1995) and home videos and pictures from adult participants’ childhoods (Rieger et al., 2008; Watts et al., 2018), most of this research has been conducted in Euro-American cultures. Thus, future non-Euro-American research should assess the relationship between childhood behaviors and adulthood sexual orientation using longitudinal measures to further assess whether the expression of sex-atypical behavior in childhood is a cross-cultural correlate of adulthood same-sex sexuality.

Lastly, although the present sample included various *pheet thii saam* groups, subgroups exist within these categories that were not captured in the present study. These include, for example, gay kings, queens, and quings, which take active/insertive, receptive, and versatile roles during sex, respectively, as well as one-way *toms* who primarily take the active/insertive role and two-way *toms* who are versatile (e.g., Ojanen, 2009). Some prior Euro-American research has shown, for example, that gay men who take a receptive role during sex recall greater childhood sex-atypicality than those who take an insertive role (e.g., Swift-Gallant et al., 2017). Thus, including these subcategories in future Thai studies could provide further insights into gender-role expression variations linked with sex, gender, and sexuality.

### Conclusion

The present study demonstrated that Thai gay men and *sao praphet song* exhibited elevated sex-atypical childhood behaviors and adulthood occupational preferences, with *sao praphet song* exhibiting even greater sex-atypicality than gay men. In doing so, this study provides further evidence that the expression of childhood and adulthood sex-atypicality are cross-cultural correlates of male androphilia from yet another culture, with gender-role presentation possibly moderating the extent to which it is expressed. In contrast, regarding female gynephilia, feminine-presenting lesbians exhibited sex-atypical childhood behaviors, but *dees* did not, and neither group reported sex-atypical adulthood occupational preferences. The expression of sex-atypical childhood behaviors *and* adulthood occupational preferences was only found among the masculine-presenting *toms*. As such, among females, gender-role presentation/gender identity might be stronger predictors of childhood and adulthood sex-atypicality than sexual orientation. Finally, the expression of childhood sex-atypical behavior was correlated with adulthood sex-atypical occupational preferences among all androphilic male and gynephilic female groups, suggesting that a developmental continuity exists between childhood and adulthood sex-(a)typicality.

## Supplementary Information

Below is the link to the electronic supplementary material.

Supplementary file1 (DOCX 49 KB)

## Data Availability

Data are available publicly at 10.5683/SP3/4JUNKF.

## Footnotes

1 In the present article, the terms “male,” “female,” and “sex” are used in reference to sex assigned at birth based on the makeup of the genitalia or sex chromosomes during fetal development or at birth. These terms are not intended to be interpreted as referring to gender. When referring to a specific group of individuals who are not cisgender—that is, transgender or gender diverse, in which birth-assigned sex and experienced gender identity do not align relative to cultural norms—the culturally relevant identity terms are used.

2 We use the term “sex-atypical” when referring to the elevated expression of female-typical behaviors among males and the elevated expression of male-typical behavior among females. Thus, the term “atypical” here is used in the statistical sense when describing members of a given sex who display elevated expression of behaviors and interests that are more typical of the other sex.

3 In Thai language, sex and gender are conflated and the words “ชาย” and “หญิง” (English transliteration: “phuchay” and “phuying”) mean both male/man and female/woman, respectively. Moreover, these terms also imply heterosexuality, so individuals who identify as male/man and female/woman are also identifying as heterosexual. For this reason, “gay” and “bi-man,” and “lesbian” and “bi-woman” were listed as separate sex/gender/sexual identity options.

4 Excluding participants who were not exclusively or predominantly androphilic and gynephilic yielded the same general pattern of results (see Supplementary Online Appendix).

5 Two a priori power analyses were conducted using G*Power 3.1.9.2 (see Faul et al., 2007) with statistical power set at the recommended .80 level (Cohen, 1988). The “Means: Difference between two independent means (two groups)” statistical test was used with the sample size allocation ratios set to .97 (i.e.,$\frac{\text{1539 lesbian women}}{\text{1583 heterosexual women}}$) and .14 (i.e.,$\frac{\text{697 lesbian women}}{\text{5053 heterosexual women}}$) based on the relevant sample sizes from Bailey and Zucker’s (1995) and Lippa’s (2005a) meta-analyses, respectively.
