# Correction: Sexual and Gender Diversity in Thailand: Associations with Recalled Childhood Sex-Typed Behavior and Adulthood Occupational Preferences

**DOI:** 10.1007/s10508-025-03265-5

**Published:** 2025-08-13

**Authors:** Francisco R. Gómez Jiménez, Ashley K. Dhillon, Doug P. VanderLaan

**Affiliations:** 1 Centre for Culture and Evolution, Brunel University of London, Uxbridge, UB8 3PH UK; 2 Department of Psychology, University of Toronto Mississauga, Mississauga, ON L5L 1C6 Canada; 3 Child and Youth Psychiatry, Centre for Addiction and Mental Health, Toronto, ON Canada

**Correction to: Archives of Sexual Behavior** 10.1007/s10508-025-03121-6

Due to an error during production, incorrect “Coome et al., 2018” reference citations were provided on the third page of the article to the following reference:

Coome, L. A., Skorska, M. N., & VanderLaan, D. P. (2020). Direct reproduction and sexual orientation and gender diversity in Thailand. *Archives of Sexual Behavior, 49*, 2449–2460. 10.1007/s10508-020-01830-8.

The citations of this reference in the text have been corrected to “Coome et al., 2020”, and the entry for this reference has been added to the References.

The original article has been corrected.

