# Supplementary material for: Sexual and Gender Diversity in Thailand: Associations with Recalled Childhood Sex-Typed Behavior and Adulthood Occupational Preferences
Source: Arch Sex Behav. 2025 Apr 24;54(8):2839–57. doi: 10.1007/s10508-025-03121-6 (PMC12484278; doi:10.1007/s10508-025-03121-6)
Supplement: Supplementary file 1 — Supplementary file1 (DOCX 49 KB) [file 10508_2025_3121_MOESM1_ESM.docx]

**Supplementary Online Appendix for “Sexual and Gender Diversity in Thailand: Associations with Recalled Childhood Sex-Typed Behavior and Adulthood Occupational Preferences”**

**Appendix A. Supplementary analyses among exclusively or predominantly androphilic and gynephilic participants**

We re-ran the analyses reported in the main text with more exclusive inclusion criteria to determine whether the results were significantly influenced by the inclusion of participants whose sexual attraction was not directed exclusively or predominantly towards the expected groups. As reported in the main text, sexual attraction was assessed by asking participants to report their sexual attraction during the last 12 months towards men (including heterosexual, bisexual, and/or gay men), women (including heterosexual, bisexual, and/or lesbian women, as well as *dees*), *sao praphet song*, and *toms*. Participants responded using a seven-point scale ranging from 0 (*none of my sexual attractions*) to 6 (*all of my sexual attractions*). Responses to the four sexual attraction targets needed to sum to 6 to represent all sexual attractions experienced over the past year. Participants were categorized as exclusively/predominantly androphilic if their sexual attraction towards men and *sao praphet song*, both of which are male at birth, added up to at 5 or 6, and as exclusively/predominantly gynephilic if their sexual attraction towards women and *toms*, both of which are female at birth, added up to 5 or 6. Heterosexual men, *dees,* lesbian women, and *toms* who were not categorized as exclusively/predominantly gynephilic, and heterosexual women, gay men, and *sao praphet song* who were not categorized as exclusively/predominantly androphilic were excluded. Relative to the sample size in the main text, this led to the exclusion of 11 heterosexual men (*n* = 259), 20 gay men (*n* = 179), 4 *sao praphet song* (*n* = 162), 9 *toms* (*n* = 65), 8 lesbian women (*n* = 48), 16 *dees* (*n* = 133), and 38 heterosexual women (*n* = 242; see Table S1).

The statistical analyses reported in Tables S1 to S7 followed the same procedures described in the main text, with the critical alpha set at .05 as in prior similar studies to facilitate comparability of findings across cultures. The discussion below only focuses on the results that differed from those reported in the main text, which were only found within the post hoc pairwise comparisons for the recalled childhood sex-typed behaviours and reported adulthood occupational preferences and the regression for the female participants. In contrast to the main analysis, no differences in recalled childhood male-typical behaviors were found between lesbian women and *sao praphet song*, lesbian women and *dees*, and *dees* and heterosexual women. While we did not have any specific predictions regarding group differences between lesbian women, *sao praphet song*, and *dees*, we did expect *dees* to exhibit greater sex-atypicality than heterosexual women. Thus, the results from both the main and supplementary analyses were inconsistent with this prediction.

With respect to the pairwise comparisons for the reported occupational preferences in adulthood, gay men had greater MF-Occ scores than heterosexual women. While this result was not significant in the main analysis, it is consistent with the overall pattern observed and discussed in the main text that Thai gay men appeared to be less male-typical than heterosexual women. Moreover, it is worth noting that same pairwise comparison in the main text was close to significance (*p* = .051). Thus, the implication of our findings remains unchanged.

With respect to the regression for female participants, Model 2 indicated that compared with heterosexual women, being in the *dee* (*β* = 0.08, *p* < .001) group was an independent predictor of higher MF-Occ (Table S7), which was not the case in the same analysis presented in the main text. The difference in the pattern of results when using more exclusive inclusion criteria might suggest that when compared with cisgender women who are exclusively/predominantly androphilic, females who identify as *dees* and are exclusively/predominantly gynephilic tend to have a higher preference for female-typical occupations that is independent of any difference in childhood-sex typed behavior that exist between the two groups. Nevertheless, the fact that heterosexual and women and *dees* did not differ with respect to recalled childhood sex-atypical behaviour and adulthood occupational preferences in the post hoc pairwise comparisons when using a more conservative critical alpha (Table S4) suggests that the significant effect found for the Heterosexual Women vs *Dees* predictor variable could have been a Type 1 error. Thus, we err on the side of caution when interpreting this effect. Overall, then, the pattern of results found when excluding participants who were not exclusively or predominantly androphilic and gynephilic were largely the same as those reported in the main text.

**Table S1** Descriptive statistics for sexual attractions during the previous year by group.

| Group | *n* | Sexual Attraction Target *M* (*SD*) | | | |
| --- | --- | --- | --- | --- | --- |
|  |  | Men | Women | *Toms* | *Sao Praphet Song* |
| Heterosexual Men | 259 | 0.01 (0.09) | 5.83 (0.45) | 0.08 (0.34) | 0.08 (0.27) |
| Gay Men | 179 | 5.70 (0.95) | 0.08 (0.27) | 0.02 (0.13) | 0.21 (0.85) |
| *Sao Praphet Song* | 162 | 5.93 (0.28) | 0.02 (0.14) | 0.04 (0.19) | 0.01 (0.11) |
| *Toms* | 165 | 0.03 (0.17) | 5.85 (0.57) | 0.07 (0.47) | 0.05 (0.22) |
| Lesbian Women | 48 | 0.23 (0.43) | 4.71 (1.68) | 1.02 (1.58) | 0.04 (0.20) |
| *Dees* | 133 | 0.17 (0.38) | 0.43 (0.96) | 5.38 (1.09) | 0.02 (0.12) |
| Heterosexual Women | 242 | 5.87 (0.37) | 0.06 (0.23) | 0.06 (0.23) | 0.01 (0.11) |

For all sexual attraction questions, the possible range for responses was from 0, representing “none of my sexual attractions” to 6, representing “all of my sexual attractions.” Each participant’s responses to the sexual attraction questions had to sum to 6. The target “men” included heterosexual, bisexual, and/or gay men. The target “women” included heterosexual, bisexual, and/or lesbian women, as well as *dees*

**Table S2** Correlations between descriptive and sex-typed variables.

| Sex-typed variables | Age | | Level of Education | | | Weekly Income | |
| --- | --- | --- | --- | --- | --- | --- | --- |
|  | Pearson’s *r* | *p*-value | Point biserial *r_pb_* | *p*-value | Point biserial *r_pb_* | | *p*-value |
| CFTB | .035 | .224 | -.008 | .784 | -.010 | | .723 |
| CMTB | .017 | .552 | .040 | .172 | .025 | | .390 |
| CSAB | **-.084** | **.004** | -.020 | .492 | -.008 | | .772 |
| FTOP | **.072** | **.013** | **.064** | **.028** | .034 | | .243 |
| MTOP | **.108** | **<.001** | .045 | .117 | .029 | | .325 |
| MF-Occ | -.038 | .194 | .006 | .829 | -.000 | | .997 |

*Abbreviations*. CFTB, childhood female-typical behavior; CMTB, childhood male-typical behavior; CSAB, childhood sex-atypical behavior; FTOP, female-typical occupational preferences; MTOP, male-typical occupational preferences; MF-Occ, male-versus-female-typical occupational preferences.

*Note*. Significant correlations are bolded. Results that differed from the analyses reported in the main text were highlighted in yellow.

**Table S3** Comparison of childhood sex-typed behavior scores and occupational preferences scores by group.

|  | Heterosexual Men  (*n* = 259) | | Gay Men  (*n* = 179) | | *Sao Praphet Song*  (*n* = 162) | | *Toms*  (*n* = 165) | | Lesbian Women  (*n* = 48) | | *Dees*  (*n* = 133) | | Heterosexual Women  (*n* = 242) | | One-way ANOVA^a^ | | | |
| --- | --- | --- | --- | --- | --- | --- | --- | --- | --- | --- | --- | --- | --- | --- | --- | --- | --- | --- |
|  | *M* | *SD* | *M* | *SD* | *M* | *SD* | *M* | *SD* | *M* | *SD* | *M* | *SD* | *M* | *SD* | *F* | *df* | *p* | *η*² |
| CFTB | 199 | 0.62 | 3.49 | 0.98 | 4.50 | 0.60 | 2.71 | 0.84 | 3.77 | 1.08 | 4.37 | 0.63 | 4.45 | 0.59 | 494.91 | 356.24 | <.001 | 0.65 |
| CMTB | 4.43 | 0.61 | 3.20 | 0.89 | 2.40 | 0.92 | 3.94 | 0.72 | 2.84 | 0.94 | 2.41 | 0.91 | 2.67 | 0.90 | 222.89 | 357.29 | <.001 | 0.48 |
| CSAB | 1.55 | 0.81 | 4.29 | 1.34 | 6.10 | 1.14 | 5.23 | 1.29 | 3.08 | 1.80 | 2.04 | 1.19 | 2.23 | 1.04 | 509.77 | 352.65 | <.001 | 0.69 |
| FTOP | 3.60 | 0.93 | 4.65 | 1.00 | 4.77 | 1.07 | 3.89 | 1.00 | 4.51 | 1.17 | 4.64 | 0.93 | 4.70 | 0.91 | 48.01 | 360.88 | <.001 | 0.19 |
| MTOP | 4.66 | 1.05 | 3.07 | 1.11 | 2.56 | 0.99 | 4.31 | 1.03 | 3.50 | 1.15 | 3.23 | 1.04 | 3.58 | 1.19 | 94.49 | 365.25 | <.001 | 0.31 |
| MF-Occ | -1.05 | 1.17 | 1.58 | 1.32 | 2.20 | 1.17 | -0.42 | 1.13 | 1.00 | 1.38 | 1.41 | 1.09 | 1.13 | 1.07 | 196.07 | 361.96 | <.001 | 0.51 |

*Abbreviations*. CFTB, childhood female-typical behavior; CMTB, childhood male-typical behavior; CSAB, childhood sex-atypical behavior; FTOP, female-typical occupational preferences; MTOP, male-typical occupational preferences; MF-Occ, male-versus-female-typical occupational preferences.

*Note*. Values for the MF-Occ scores ranged from -6 to 6, with positive scores representing higher female-typical behavior and negative scores representing greater male-typical behavior. Values for CSAB scores ranged from 0 to 8, with higher scores representing greater sex-atypical behavior. Results that differed from the analyses reported in the main text were highlighted in yellow.

^a^ Due to differences in sample sizes and significant Levene’s tests for equality of variance (*p* < .05), all one-way ANOVAs were performed using Welch tests.

**Table S4** Post hoc pairwise comparisons of childhood sex-typed behavior scores.

| Group Comparison | CFTB | | CMTB | | CSAB | |
| --- | --- | --- | --- | --- | --- | --- |
|  | *p* | *Cohen’s d* (95% CI) | *p* | *Cohen’s d* (95% CI) | *p* | *Cohen’s d* (95% CI) |
| Heterosexual Men - Gay Men | **<.001** | **-1.83 (-2.09, -1.56)** | **<.001** | **1.63 (1.37, 1.87)** | **<.001** | **-2.47 (-2.73, -2.19)** |
| Heterosexual Men - *Sao Praphet Song* | **<.001** | **-4.12 (-4.62, -3.68)** | **<.001** | **2.62 (2.22, 3.00)** | **<.001** | **-4.58 (-5.09, -4.07)** |
| Heterosexual Men - *Toms* | **<.001** | **-0.98 (-1.18, -0.77)** | **<.001** | **0.74 (0.53, 0.95)** | **<.001** | **-3.82 (-4.23, -3.43)** |
| Heterosexual Men - *Lesbian Women* | **<.001** | **-2.02 (-2.52, -1.56)** | **<.001** | **2.01 (1.57, 2.45)** | **<.001** | **-1.09 (-1.42, -0.72)** |
| Heterosexual Men - *Dees* | **<.001** | **-3.80 (-4.30, -3.35)** | **<.001** | **2.62 (2.25, 3.04)** | **<.001** | **-0.47 (-0.68, -0.26)** |
| Heterosexual Men - Heterosexual Women | **<.001** | **-4.06 (-4.49, -3.66)** | **<.001** | **2.3 (2.03, 2.57)** | **<.001** | **-0.72 (-0.91, -0.54)** |
| Gay Men - *Sao Praphet Song* | **<.001** | **-1.24 (-1.46, -1.01)** | **<.001** | **0.88 (0.61, 1.10)** | **<.001** | **-1.45 (-1.69, -1.17)** |
| Gay Men - *Toms* | **<.001** | **0.85 (0.62, 1.10)** | **<.001** | **-0.92 (-1.16, -0.71)** | **<.001** | **-0.77 (-1.00, -0.55)** |
| Gay Men - Lesbian Women | .678 | -0.27 (-0.62, 0.05) | .244 | 0.39 (0.05, 0.71) | **<.001** | **0.77 (0.41, 1.15)** |
| Gay Men - *Dees* | **<.001** | **-1.07 (-1.30, -0.84)** | **<.001** | **0.87 (0.63, 1.13)** | **<.001** | **1.78 (1.48, 2.05)** |
| Gay Men - Heterosexual Women | **<.001** | **-1.18 (-1.37, -0.97)** | **<.001** | **0.59 (0.39, 0.79)** | **<.001** | **1.72 (1.48, 1.95)** |
| *Sao Praphet Song* - *Toms* | **<.001** | **2.45 (2.07, 2.80)** | **<.001** | **-1.87 (-2.17, -1.55)** | **<.001** | **0.78 (0.54, 1.01)** |
| *Sao Praphet Song* - Lesbian Women | **<.001** | **0.83 (0.52, 1.21)** | .072 | -0.48 (-0.81, -0.10) | **<.001** | **2.00 (1.54, 2.53)** |
| *Sao Praphet Song* - *Dees* | .625 | 0.20 (-0.04, 0.41) | 1.00 | -0.01 (-0.24, 0.21) | **<.001** | **3.49 (3.06, 3.97)** |
| *Sao Praphet Song* - Heterosexual Women | .979 | 0.09 (-0.11, 0.29) | **.049** | **-0.30 (-0.50, -0.08)** | **<.001** | **3.54 (3.13, 3.9)** |
| *Toms* - Lesbian Women | **<.001** | **-1.09 (-1.47, -0.69)** | **<.001** | **1.31 (0.91, 1.65)** | **<.001** | **1.45 (0.97, 1.89)** |
| *Toms* - *Dees* | **<.001** | **-2.24 (-2.57, -1.86)** | **<.001** | **1.87 (1.54, 2.18)** | **<.001** | **2.80 (2.42, 3.16)** |
| *Toms* - Heterosexual Women | **<.001** | **-2.39 (-2.73, -2.08)** | **<.001** | **1.56 (1.31, 1.80)** | **<.001** | **2.82 (2.53, 3.06)** |
| Lesbian Women - *Dees* | **.009** | **-0.69 (-1.03, -0.32)** | .099 | 0.47 (0.10, 0.82) | **.008** | **0.68 (0.33, 1.04)** |
| Lesbian Women - Heterosexual Women | **.002** | **-0.78 (-1.14, -0.46)** | .910 | 0.18 (-0.15, 0.49) | **.039** | **0.58 (0.19, 0.95)** |
| *Dees* – Heterosexual Women | .940 | -0.12 (-0.33, 0.11) | .104 | -0.29 (-0.50, -0.07) | .707 | -0.17 (-0.40, 0.05) |

*Abbreviations*. CFTB, childhood female-typical behavior; CMTB, childhood male-typical behavior; CSAB, childhood sex-atypical behavior

*Note*. All pairwise comparisons were conducted using the Games Howell procedure due to differences in sample size and significant Levene’s tests for equality of variance (*p* < .05). Significant pairwise comparisons are bolded. Results that differed from the analyses reported in the main text were highlighted in yellow.

**Table S5** Post hoc pairwise comparisons of occupational preferences scores.

| Group Comparison | FTOP | | MTOP | | MF-Occ | |
| --- | --- | --- | --- | --- | --- | --- |
|  | *p* | *Cohen’s d* (95% CI) | *p* | *Cohen’s d* (95% CI) | *p* | *Cohen’s d* (95% CI) |
| Heterosexual Men - Gay Men | **<.001** | **-1.08 (-1.28, -0.87)** | **<.001** | **1.47 (1.22, 1.71)** | **<.001** | **-2.11 (-2.34, -1.82)** |
| Heterosexual Men - *Sao Praphet Song* | **<.001** | **-1.16 (-1.41, -0.93)** | **<.001** | **2.05 (1.78, 2.34)** | **<.001** | **-2.78 (-3.03, -2.49)** |
| Heterosexual Men - *Toms* | .059 | -0.29 (-0.51, -0.09) | **.015** | **0.33 (0.13, 0.52)** | **<.001** | **-0.55 (-0.75, -0.34)** |
| Heterosexual Men - *Lesbian Women* | **<.001** | **-0.85 (-1.27, -0.46)** | **<.001** | **1.05 (0.68, 1.41)** | **<.001** | **-1.61 (-1.99, -1.24)** |
| Heterosexual Men - *Dees* | **<.001** | **-1.11 (-1.34, -0.84)** | **<.001** | **1.37 (1.16, 1.58)** | **<.001** | **-2.18 (-2.42, -1.96)** |
| Heterosexual Men - Heterosexual Women | **<.001** | **-1.19 (-1.36, -0.99)** | **<.001** | **0.96 (0.79, 1.16)** | **<.001** | **-1.95 (-2.14, -1.74)** |
| Gay Men - *Sao Praphet Song* | .928 | -0.12 (-0.34, 0.10) | **<.001** | **0.48 (0.24, 0.69)** | **<.001** | **-0.50 (-0.71, -0.30)** |
| Gay Men - *Toms* | **<.001** | **0.76 (0.53, 0.96)** | **<.001** | **-1.16 (-1.40, -0.89)** | **<.001** | **1.62 (1.39, 1.85)** |
| Gay Men - Lesbian Women | .989 | 0.13 (-0.19, 0.46) | .238 | -0.39 (-0.73, -0.08) | .150 | 0.42 (0.11, 0.76) |
| Gay Men - *Dees* | 1.00 | 0.00 (-0.21, 0.23) | .832 | -0.15 (-0.37, 0.08) | .880 | 0.14 (-0.08, 0.35) |
| Gay Men - Heterosexual Women | .997 | -0.06 (-0.27, 0.13) | **<.001** | **-0.44 (-0.64, -0.24)** | **.004** | **0.38 (0.17, 0.57)** |
| *Sao Praphet Song* - *Toms* | **<.001** | **0.85 (0.60, 1.10)** | **<.001** | **-1.72 (-2.01, -1.42)** | **<.001** | **2.28 (2.01, 2.49)** |
| *Sao Praphet Song* - Lesbian Women | .810 | 0.23 (-0.09, 0.53) | **<.001** | **-0.87 (-1.23, -0.53)** | **<.001** | **0.94 (0.61, 1.30)** |
| *Sao Praphet Song* - *Dees* | .928 | 0.13 (-0.12, 0.36) | **<.001** | **-0.66 (-0.9, -0.42)** | **<.001** | **0.70 (0.47, 0.97)** |
| *Sao Praphet Song* - Heterosexual Women | .995 | 0.07 (-0.14, 0.28) | **<.001** | **-0.92 (-1.14, -0.70)** | **<.001** | **0.96 (0.73, 1.16)** |
| *Toms* - Lesbian Women | **.023** | **-0.57 (-0.94, -0.17)** | **<.001** | **0.74 (0.36, 1.10)** | **<.001** | **-1.13 (-1.49, -0.76)** |
| *Toms* - *Dees* | **<.001** | **-0.78 (-1.02, -0.52)** | **<.001** | **1.04 (0.78, 1.29)** | **<.001** | **-1.65 (-1.87, -1.42)** |
| *Toms* - Heterosexual Women | **<.001** | **-0.85 (-1.05, -0.63)** | **<.001** | **0.65 (0.45, 0.86)** | **<.001** | **-1.40 (-1.60, -1.24)** |
| Lesbian Women - *Dees* | .992 | -0.13 (-0.50, 0.21) | .780 | 0.25 (-0.11, 0.61) | .531 | -0.33 (-0.66, 0.04) |
| Lesbian Women - Heterosexual Women | .930 | -0.19 (-0.56, 0.11) | 1.00 | -0.06 (-0.40, 0.25) | .997 | -0.10 (-0.45, 0.22) |
| *Dees* – Heterosexual Women | .996 | -0.07 (-0.29, 0.14) | .057 | -0.31 (-0.53, -0.12) | .191 | 0.26 (0.04, 0.48) |

*Abbreviations*. FTOP, female-typical occupational preferences; MTOP, male-typical occupational preferences; MF-Occ, male-versus-female-typical occupational preferences.

*Note*. All pairwise comparisons were conducted using the Games Howell procedure due to differences in sample size and significant Levene’s tests for equality of variance (*p* < .05). Significant pairwise comparisons are bolded. Results that differed from the analyses reported in the main text were highlighted in yellow.

**Table S6** Linear regression predicting male- versus female-typical occupational preferences scores based on gender and childhood sex-atypical behavior scores for birth-assigned male groups.

|  |  | Male-versus-female-typical occupational preferences | | | | | |
| --- | --- | --- | --- | --- | --- | --- | --- |
|  |  | *B* | 95% CI | SE | *β* | *t* | *p* |
| *Model* | *Predictor* |  |  |  |  |  |  |
| 1 | Childhood sex-atypical behavior | 0.65 | 0.60, 0.69 | 0.02 | 0.75 | 27.43 | <.001 |
|  |  |  |  |  |  |  |  |
| 2 | Childhood sex-atypical behavior | 0.32 | 0.23, 0.41 | 0.04 | 0.37 | 7.24 | <.001 |
|  | Heterosexual Men vs Gay Men | 1.75 | 1.42, 2.08 | 0.17 | 0.42 | 10.60 | <.001 |
|  | Heterosexual Men vs *Sao Praphet Song* | 1.81 | 1.35, 2.26 | 0.23 | 0.42 | 7.80 | <.001 |

Model 1: *R*^2^ = .557; Adjusted *R*^2^ = .556; *F*(1, 598) = 752.30, *p* < .001.

Model 2: *R*^2^ = .628; Adjusted *R*^2^ = .626; *F* (3, 596) =334.70, *p* < 001.

∆*R*^2^ = .070, *F* (2, 596) = 56.29, *p* <.001.

*Note*. For both dummy variables, heterosexual men were coded as 0. In Heterosexual Men vs Gay Men, *sao praphet song* were coded as 0 and gay men as 1. In Heterosexual Men vs *Sao Praphet Song*, gay men were coded as 0 and *sao praphet song* as 1. Results that differed from the analyses reported in the main text were highlighted in yellow.

**Table S7** Linear regression predicting male- versus female-typical occupational preferences scores based on gender and childhood sex-atypical behavior scores for birth assigned female groups.

|  |  | Male-versus-female-typical occupational preferences | | | | | |
| --- | --- | --- | --- | --- | --- | --- | --- |
|  |  | *B* | 95% CI | SE | *β* | *t* | *p* |
| *Model* | *Predictor* |  |  |  |  |  |  |
| 1 | Childhood sex-atypical behavior | -0.40 | -0.45, -0.35 | 0.03 | -0.53 | -15.31 | <.001 |
|  |  |  |  |  |  |  |  |
| 2 | Childhood sex-atypical behavior | -0.21 | -0.29, -0.13 | 0.04 | -0.28 | -5.42 | <.001 |
|  | Heterosexual Women vs *Dees* | 0.24 | 0.01, 0.47 | 0.12 | 0.08 | 2.05 | .041 |
|  | Heterosexual Women vs Lesbian Women | 0.06 | -0.29, 0.40 | 0.18 | 0.01 | 0.32 | .747 |
|  | Heterosexual Women vs *Toms* | -0.92 | -1.23, -0.60 | 0.16 | -0.31 | -5.71 | <.001 |

Model 1: *R*^2^ = .286; Adjusted *R*^2^ = .284; *F*(1, 586) = 234.3, *p* < .001.

Model 2: *R*^2^ = .339; Adjusted *R*^2^ = .334; *F* (4, 583) = 74.61, *p* < .001.

∆*R*^2^ = .053, *F* (3, 583) = 15.56, *p* < .001

*Note*. For the three dummy variables, heterosexual women were coded as 0. In Heterosexual Women vs *Dees*, lesbian women and *toms* were coded as 0 and *dees* as 1. In Heterosexual Women vs Lesbian Women, *dees* and *toms* were coded as 0 and lesbian women as 1. In Heterosexual Women vs *Toms, dees* and lesbian women were coded as 0 and *toms* as 1. Results that differed from the analyses reported in the main text were highlighted in yellow.
